# New 19-Residue Peptaibols from *Trichoderma* Clade Viride

**DOI:** 10.3390/microorganisms6030085

**Published:** 2018-08-12

**Authors:** Tamás Marik, Chetna Tyagi, Gordana Racić, Dávid Rakk, András Szekeres, Csaba Vágvölgyi, László Kredics

**Affiliations:** 1 Department of Microbiology, Faculty of Science and Informatics, University of Szeged, Közép fasor 52, H-6726 Szeged, Hungary; mariktamas88@gmail.com (T.M.); cheta231@gmail.com (C.T.); rakkdavid@gmail.com (D.R.); andras.j.szekeres@gmail.com (A.S.); csaba@bio.u-szeged.hu (C.V.); 2 Doctoral School in Biology, Faculty of Science and Informatics, University of Szeged, H-6726 Szeged, Hungary; 3 Faculty of Environmental Protection, Educons University, Vojvode Putnika 87, 21208 Sremska Kamenica, Serbia; gordana.racic84@gmail.com

**Keywords:** *Trichoderma*, Viride clade, peptaibol, liquid chromatography, mass spectrometry, bioactivity

## Abstract

*Trichoderma koningiopsis* and *T. gamsii* belong to clade *Viride* of *Trichoderma*, the largest and most diverse group of this genus. They produce a wide range of bioactive secondary metabolites, including peptaibols with antibacterial, antifungal, and antiviral properties. The unusual amino acid residues of peptaibols, i.e., α-aminoisobutyric acid (Aib), isovaline (Iva), and the C-terminal 1,2-amino alcohol make them unique among peptides. In this study, the peptaibiomes of *T. koningiopsis* and *T. gamsii* were investigated by HPLC-ESI-MS. The examined strains appeared to produce 19-residue peptaibols, most of which are unknown from literature, but their amino acid sequences are similar to those of trikoningins, tricholongins, trichostrigocins, trichorzianins, and trichorzins. A new group of peptaibols detected in *T. koningiopsis* are described here under the name “Koningiopsin”. Trikoningin KA V, the closest peptaibol compound to the peptaibols produced by these two strains, was selected for structural investigation by short MD simulation, which revealed that many residues show high preference for left handed helix formation. The bioactivity of the peptaibol mixtures produced by *T. koningiopsis* and *T. gamsii* was tested on agar plates against bacteria, yeasts, and filamentous fungi. The results revealed characteristic differences in bioactivities towards the different groups of target microorganisms, which can be explained with the differences in their cell wall structures.

## 1. Introduction

Within the filamentous fungal genus *Trichoderma* (Ascomycota, Hypocreales, Hypocreaceae) comprising more than 250 species [1], clade Viride forms one of the largest and most diverse groups. The majority of *Trichoderma* species were described after the year 2000, only a few species were initially included in the genus [2,3]. Bissett [4] proposed to include *H. rufa/T. viride* and its relatives in *Trichoderma* section Trichoderma along with *T. koningii* Oudem. and *T. atroviride* P. Karst. The monophyly of this group, earlier referred to as *Trichoderma* section Trichoderma [5] and recently as clade Viride, was confirmed after DNA sequence analysis of the internal transcribed spacers 1 and 2 (ITS 1 and 2), as well as fragments of the actin (*act*), calmodulin (*cal*) and translation elongation factor 1α (*tef*1) genes [6]. Since the work of Lieckfeldt et al. [7], many additional species and cultures referable to clade Viride were obtained and the taxonomy of this clade has also been revised [8]. Species in this clade can be isolated from very diverse sources with a wide geographic distribution [9] and they were also reported as beneficial organisms of industrial, agricultural, and medicinal fields [10,11].

*Trichoderma* species are known to produce a broad range of bioactive secondary metabolites with antibacterial, antifungal, and antiviral properties [12]. Among them, peptaibols are non-ribosomal peptides containing unusual amino acid residues like α-aminoisobutyric acid (Aib), as well as isovaline (Iva), and a C-terminal 1,2-amino alcohol (e.g., Leuol, Valol, Pheol, Tyrol, Ileol, Alaol, or Prool) [13,14]. The biosynthesis of these peptides significantly differs from the ribosomal pathway: they are assembled by large, modular enzymes known as non-ribosomal peptide synthetases (NRPS) [15,16]. The amino acid sequence of peptaibols usually appears as a short linear helical structure, therefore several molecules need to aggregate for the formation of ion channels, which are able to cause membrane damage in lipid bilayers [17].

The information available in the literature about the peptaibol profiles of *T. koningiopsis* and *T. gamsii* is limited. Peptaibols produced by *T. koningiopsis* were previously identified as trikoningin KA V, together with other 11-residue compounds, trikoningin KB I and KB II [18]. To the best of our knowledge, other peptaibol compounds produced by *T. koningiopsis* were not previously reported in the literature. *Trichoderma gamsii* is a widespread species of the genus, also known as an endophyte of the traditional Chinese medicinal plant *Panax notoginseng*. Although the investigation of peptaibol production was not yet carried out for this species, *T. gamsii* was shown to produce numerous secondary metabolites including cytochalasans [19,20,21,22], the spiro-cytochalasan trichodermone [21], trichoderamides A and B originating from the PKS-NRPS hybrid pathway [23], trichodenols A and B [23], trichoderpyrone [24], as well as volatile organic compounds like dimethyl disulfide, dibenzofuran, methanethiol, and ketones [25]. Among the detected cytochalasans (trichalasins A, B, C, D, E, F, G, H, aspochalasins D, I, J, K, M, P, and aspergillin PZ), aspochalasins D and I displayed weak inhibitory activity against the HeLa cancer cell line [19,20], trichalasin G proved to be modestly inhibitory to the human cancer cell line MDA-MB-231 [22], while trichoderpyrone displayed weak cytotoxic activities against A549, HepG2, and HeLa cancer cell lines [24].

The aim of the present study was to investigate the peptaibiomes of these two species and to characterise their antibiotic activity against a broad spectrum of microorganisms.

## 2. Materials and Methods

### 2.1. Strains and Culture Conditions

All strains used in this study are deposited in the Szeged Microbiology Collection (SZMC; www.szmc.hu). The *Trichoderma* strains selected for the investigation of their peptaibol production, *T. gamsii* SZMC 1656 and *T. koningiopsis* SZMC 12500, were identified by Nucleotide BLAST analysis (https://blast.ncbi.nlm.nih.gov/Blast.cgi) of a part of the *tef*1 gene amplified according to Castagnoli et al. [26], and proved to be very closely related to *T. gamsii* S582 (GenBank: KJ665495) and *T. koningiopsis* UNISS 17b-36a (GenBank: EF488124), respectively. Both *Trichoderma* strains were maintained on malt extract agar (MEA) supplemented with yeast extract (0.25 g L^−1^ yeast extract, 0.5 g L^−1^ malt extract, 1 g L^−1^ glucose, 2 g L^−1^ agar in distilled water; pH 6.5). To increase peptaibol production, strains were inoculated to large (40 × 40 cm) plates containing malt extract agar (MEA) medium (30 g L^−1^ malt extract, 3 g L^−1^ soy peptone, 15 g L^−1^ agar in distilled water; pH 5.5) and incubated for 7 days at 25 °C. Bacteria (*Escherichia coli* SZMC 0582, *Micrococcus luteus* SZMC 0264 *Pseudomonas aeruginosa* SZMC 0568, *Staphylococcus aureus* SZMC 0579) and fungi (*Candida boidinii* SZMC 0673, *Kluyveromyces lactis* SZMC 0683, *Saccharomyces cerevisiae* SZMC 0425, *Schizosaccharomyces pombe* SZMC 0142, *Alternaria alternata* SZMC 16085, *Fusarium solani* species complex SZMC 11467, *Rhizoctonia solani* SZMC 6252J, *Phoma cucurbitacearum* SZMC 16088, *T. aggressivum* f. *europaeum* SZMC 1811, *T. pleuroti* SZMC 12454, *T. koningiopsis* SZMC 12500, *T. gamsii* SZMC 1656) involved in the bioactivity tests were maintained on LB (Luria-Bertani) agar medium (10 g L^−1^ tryptone, 5 g L^−1^ yeast extract, 10 g L^−1^ NaCl and 20 g L^−1^ agar-agar in distilled water; pH 7) and MEA completed with yeast extract (see above), respectively. 

### 2.2. Peptaibol Extraction

After 7 days of incubation on MEA medium, mycelium of the cultures was harvested from the plates and collected. Then, 300 mL chloroform/methanol 2/1 (*v*/*v*) solution was added and the mixture was shaken for 2 h. The lower phase was collected and evaporated to dryness (IKA RV 10; IKA Works, Wilmington, NC, USA), The extraction steps were repeated three times in total. After the extraction, the dry residue was dissolved in MeOH, centrifuged in a Biofuge Primo centrifuge (Heraeus, Hanau, Germany) and stored at −20 °C. The samples were diluted 100 × for HPLC-MS analysis and set to 100 µg mL^−1^ for inhibition tests.

### 2.3. Analytical Procedures

The crude peptaibol extracts were measured by using an HPLC-ESI-MS instrument with an Agilent 1100 system (Agilent Technologies, Palo Alto, CA, USA) controlled by a ChemStation software (A09.03; Agilent Technologies, Palo Alto, CA, USA). The system was equipped with a binary pump, a vacuum degasser, a µWell-plate autosampler, as well as a Jones Model 7990 Space column heater (Jones Chromatography Ltd., Lakewood, CO, USA). Peptaibol separation was carried out on Gemini NX-C18 HPLC column (150 mm × 2.0 mm, 3 µm; Phenomenex Inc., Torrance, CA, USA). Solvent A was H_2_O with 0.05% (*v*/*v*) trifluoroacetic acid (TFA), while Solvent B was acetonitrile/methanol 1/1 (*v*/*v*) with 0.05% (*v*/*v*) TFA. The flow rate was set to 0.2 mL min^−1^, the gradient program for Solvent B to 65%—0 min, 65%—5 min, 80%—45 min, 100%—70 min, 100%—75 min, 65%—76 min, 65%—81 min, the column temperature to 40 °C, and the injection volume to 5 µL. The ESI-IT-MS instrument was Varian 500 MS (Agilent Technologies, Palo Alto, CA, USA) with ESI source in positive mode at normal scan speed and controlled by the 500-MS Mass Spec module driver of the Varian Workstation software (6.6/SP1; Varian Inc., Palo Alto, CA, USA). ESI parameters were set to the following values: spray chamber temperature: 50 °C, drying gas (N_2_) pressure: 30 psi, drying gas temperature: 350 °C, nebuliser gas (N_2_) pressure: 50 psi, needle voltage: 5704 V, spray shield voltage: 600 V. The general parameters were set as the maximum scan times at 2.78, 2 μScans averaged, data rate at 0.36 Hz and multiplier offset at 0. The ionization control parameters were set as target TIC wet at 100% and max ion time at 250,000 μsec, scan parameters as capillary voltage was set at 66 V, RF loading at 147%, while the MS scan parameters were set as low mass *m/z* at 100, high mass *m/z* at 2000. The MS^2^ measurements of selected y_7_ fragments were carried out with the following excitation storage level (*m/z*)/excitation amplitude (V) conditions: *m/z* 754.5 (204.5/2.95), *m/z* 755.5 (204.8/2.96) *m/z* 768.5 (208.0/3.00), and *m/z* 769.5 (208.3/3.00).

Based on a calibration with alamethicin standard (Sigma-Aldrich Ltd., Budapest, Hungary), the peptaibol contents of the crude extracts were also calculated.

### 2.4. Nomenclature of the Identified Peptaibols

The newly identified peptaibol compounds obtained from *T. gamsii* SZMC 1656 were named according to their elution order (I, II, … n), attached to the prefix ‘Pept’. In the case of compounds eluting close to each other and differing in their characteristic ion fragments (b_12_ and y_7_), Latin letters (a and b) are following the Roman numerals. The sequences obtained from *T. koningiopsis* SZMC 12500 were named Koningiopsins and numbered with Roman numerals (I, II, … n) based on the elution order, and the different variants are distinguished by Latin letters (a and b) as mentioned before.

### 2.5. Sequence Selection and Force Field Library Generation for Non-Standard Residues

Trikoningin KA V (TKV) with the primary structure of AcAib^1^-Gly^2^-Ala^3^-Aib^4^-Ile^5^-Gln^6^-Aib^7^-Aib^8^-Aib^9^-Ser^10^-Leu^11^-Aib^12^-Pro^13^-Val^14^-Aib^15^-Ile^16^-Gln^17^-Gln^18^-Leuol^19^ was selected for molecular dynamics studies. Aib and Leuol are non-standard (non-proteinogenic) amino acid residues in the selected sequence. The R.E.D server [27] was used for calculation of their partial charges and creating force field libraries. R.E.D stands for RESP ESP charge derive [28]. RESP (restrained electrostatic potential) was used to calculate the charges with a HF/6-311G(d) basis set and Gaussian09 as quantum mechanical program interface. For the Aib residue, two conformations, i.e., α-helix (Φ = −63.8, Ψ = −38.3) and β-sheet or C_5_ (Φ = −157.2, Ψ = 161.9) were used. These were modified based on the strategy described by Cieplak et al. [29]. A slightly different strategy was used to calculate the charges for Leuol where two molecules, ethanol and Leu, were used to form the Leuol unit. The results include the charges calculated in the molecule files and a script to make force field libraries for these forces (Supplementary Data 1). The sequence was built by supplying residue units from the scratch using “tleap” after sourcing the library files of non-standard amino acids.

### 2.6. Molecular Dynamics Simulations of Trikoningin KA V

The MD calculation was carried out with Amber14 [30] using ff14SB force field available on the NIIF server via University of Szeged. The first step was “energy minimization” to stabilize the system. The maximum number of cycles was set at 10,000 (maxcyc) with convergence criteria of 0.01. The Steepest descent algorithm was used for the first 100 cycles (ncyc) and then switched to conjugate-gradient algorithm. The energy minimization outputs were used for setting up the production run with 50,000,000 steps which correspond to 10,000 ps (frames) and therefore, 100 ns of total simulation time. The generalized born implicit solvent method was used to study this system. The whole system was maintained at 300 K using Langevin thermostat (ntt = 3, gamma ln = 1.0). The time step was set to 2 fs and no cutoff was applied for non-bonding interactions. The resultant trajectories were visualized in VMD (Visual Molecular Dynamics) [31]. Further secondary structure analysis was done by *cpptraj* [32] module of AmberTools18.

### 2.7. Testing the Inhibitory Effects of Peptaibol Extracts to Strains of Bacteria, Yeasts, and Filamentous Fungi

For inhibition tests with the bacteria, LB agar medium was incubated at 37 °C based on the method of Marik et al. [33]. The same protocol was used for the inhibition assays with yeasts and filamentous fungi by using MEA supplemented with yeast extract. Agar plugs cut from the colonies of the fungal strains were placed in the centre of the plates and holes (5 mm in diameter) were bored around in 3 cm distance from the centre of the plate. Two-fold dilution series of the 100 mg mL^−1^ crude peptaibol extracts—which were also examined for their peptaibol composition—were tested, with methanol as control. The cultures were incubated at 25 °C. Photographs were taken with a Nikon Coolpix S2600 camera at two stages, when the edge of the culture reached the control hole and when it reached the edge of the Petri-dishes. Three parallel experiments were set up to measure the inhibition zones.

## 3. Results and Discussion

### 3.1. Identification, Sequencing, and Quantitation of Peptaibol Compounds Produced by T. gamsii and T. koningiopsis

Peptaibols produced by the examined species were identified based on the protocol described by Marik et al. [34]. The sequences were determined based on the observation of the characteristic ions of the compounds ([M + Na]^+^, [M + 2Na]^2+^, b_12_ and y_7_ ion) and the retention time. The b_n_ fragments could be identified after the MS measurements, while the y_7_ fragments could only be observed after MS^2^ investigations. The characteristic mass difference Δm = 213 Da could be observed in all MS spectra, resulting from the Gln6–Aib7 bond due to its stability under the fragmentation conditions of ESI-MS [35,36,37]. The b_14_ fragments were not detected on the MS spectra after the Aib–Pro bond between positions 12 and 13, furthermore, the y7-AA(19-15) ions could also not be detected with MS^2^, therefore the amino acids in positions 14 and 15 (Vxx14-Aib15) were predicted based on the Comprehensive Peptaibotics Database showing the frequent presence of the Aib-Pro-Vxx-Aib motif in this region [14]. The sequences of the compounds identified from the two examined strains and listed in Table 1 and Table 2 are derived from de novo MS-based sequencing. As no amino acid analysis has been performed, a discrimination between isobaric amino acids was not possible. The diagnostic fragment ions of the peptaibols found in this study are shown in Tables S1–S4, presented according to Röhrich et al. [38]. The peptaibols produced by *T. gamsii* SZMC 1656 strain (Table 1, Figures S1, S3 and S5) proved to be completely different from the ones detected in the extract of *T. koningiopsis* SZMC 12500 (Table 2, Figures S2, S4 and S5). The main differences between the peptaibols of the two species could be identified in 4 positions of their sequences. In the 2nd position, peptaibols produced by *T. gamsii* SZMC 1656 contain Gly or Ala in Pept-X, -XI, and -XII, while only Ala was observed in this position in the peptaibol sequences identified from *T. koningiopsis* SZMC 12500. Another difference is at the 5th residue of the sequences, where mostly Lxx (Leu/Ile), in some cases Vxx (Val/Iva) was identified in the sequences of *T. gamsii* SZMC 1656, while the compounds of *T. koningiopsis* SZMC 12500 exhibited mostly Aib at this position. The third main difference between the sequences of the two examined species was observed at the 9th position, where mostly Aib was identified in the peptaibols produced by *T. gamsii* SZMC 1656, while those from *T. koningiopsis* SZMC 12500 mostly showed Lxx and in some cases Aib. The 18th position contains Gln in the sequences of *T. gamsii* SZMC 1656, but *T. koningiopsis* SZMC 12500 produces compounds with Glu in this position causing 2 more variants of the y_7_ ion. The sequences Pept-Vb, -VIb, and -VII were matching with trikoningin KA V, though the isomeric positions of Vxx and Lxx were not identified. All other sequences proved to be new and showed similarities to the peptaibol groups of trikoningins, tricholongins, trichostrigocins, and trichorzianins. Apart from the groups shown in Table 1, the newly identified sequences also showed high similarity to trichorzin HAs, which, however, are only 18-residue peptaibols devoid of the Gln/Glu residue [39]. 

Trikoningin KA V, identified firstly from *T. koningii* [40], is a peptaibol sequence positionally isomeric with sequences Pept-Vb, -VIb, and -VII. This compound was also found to be produced by *T. koningiopsis*, along with the 11-residue lipopeptaibols trikoningin KB I and KB II [18]. Trichostrigocins were previously identified from *T. strigosum* [41,42,43] and later also from the extracts of *T. paraviridescens* and *T. trixiae* as trichostrigocin-like compounds [26]. Tricholongins were detected in *T. longibrachiatum* [44] and *T. strigosum* [41], while trichorzianins are known from *T. atroviride* [45,46,47,48]. Further 19-residue peptaibols closely related to those of the present study include hypophellins from *T. phellinicola* (syn. *Hypocrea phellinicola*), hypopulvins from *T. pulvinatum* (syn. *H. pulvinata*), gelatinosins from *T. gelatinosum* (syn. *H. gelatinosa*), voglmayrins from *T. voglmayrii* (syn. *H. voglmayrii*), minutisporins from *T. minutisporum* (syn. *H. minutispora*) and hypocitrins from *T. citrinum* (syn. *H. citrina*) [38,48,49]. This indicates that within the genus the ability to produce 19-residue peptaibols is not restricted to clade Viride of section Trichoderma, but also occurring in sections Hypocreanum (*T. phellinicola, T. pulvinatum, T. citrinum*) and Pachybasium (*T. minutisporum*), as well as in lone lineages (*T. voglmayrii, T. gelatinosum*).

In certain cases, minor differences were observed between the presently detected and the previously reported peptaibol sequences showing amino acid exchanges only at selected positions of the peptide chain (Table 1 and Table 2). Trichorzianins differ in the position 19 of the peptide chain. This position plays a critical role in the lifetime of the opened ion channel as the substitution of Pheol to Leuol/Ileol has led to increased lifetime of the open channel [50]. This was also observed previously in the case of peptaibol-formed channels, where the Pheol was substituted to Trpol in both trichorzianin B-IIIc (Trpol) and B-VII (Pheol) [51]. On the other hand, the investigation of synthetic alamethicin analogues—where all Aib residues were changed to Leu—revealed that the substitution of the C-terminal residues was not affecting the lifetime of the open channel [52]. A secondary structural study was also carried out for the purified compound trichorzianin TA VII in association with sodium dodecyl sulfate (SDS) micelles, which revealed formation of two right-handed helical segments (1–8 and 11–19) linked by a β-turn [53]. The novelty of the peptaibols produced by T. koningiopsis SZMC 12500 is in the variation of the C-terminus, which is critical in the lifetime of the ion channels. The name “Koningiopsin” was introduced for these novel compounds.

The calculated contents of the whole peptaibol molecules were 214.28 µg mL^−1^ and 101.26 µg mL^−1^ in the crude extracts of *T. gamsii* SZMC 1656 and *T. koningiopsis* SZMC 12500, respectively. In the case of *T. gamsii* SZMC 1656, Pept VIIIb and Pept VIb were the most abundant sequences of peptaibols. The sum of the amount of these two molecules was approx. 50%, while the concentration of other peptaibols remained below 10%. In the extract of *T. koningiopsis* SZMC 12500, Pept XVIIa accounted for almost half of the peptaibols produced. A new method for quantification of peptaibols based on the length of different peptides was described by Van Bohemen et al. [54]. The different length of peptaibols results in different structures, furthermore, shorter peptaibols (11–14 residues) contain more Pro leading to a structural deformation [55]. Based on the alamethicin standard, only the longer (17–20-residue) peptaibols can be quantified with high accuracy.

### 3.2. Structural Elucidation of Trikoningin KA V Based on Short Molecular Dynamics

**Figure 1:**
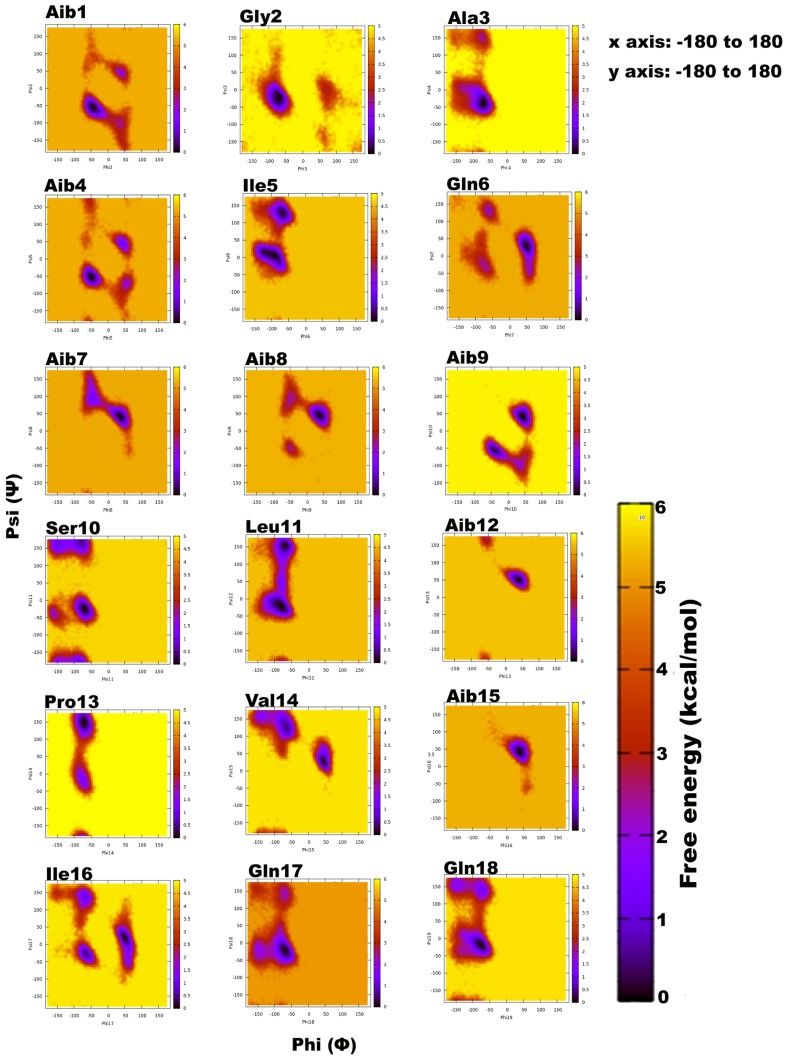
Free energy-based Ramachandran plots for each Trikoningin KA V residue during 100 ns long implicit water simulation. The x and y axes range from −180 to +180. The darkest red regions indicate toward minimum energy secondary structural regions favoured by each residue during the simulation.

Trikoningin KA V (TKV, positionally isomeric with sequences Pept-Vb, -VIb, and -VII of *T. gamsii*) is a 19-residue peptaibol with seven Aib residues constituting its sequence. Aib is an achiral residue, which has been shown to promote helix formation and can exist in both right- and left-handed helix regions on the Ramachandran plot [56,57,58,59]. To determine the propensities of each residue for a given secondary structural region on the Ramachandran plot, their relative free energies were calculated, which clearly describe an energetically favourable conformation (Figure 1). The darkest scatter populations indicate energetically preferable conformations.

**Figure 2:**
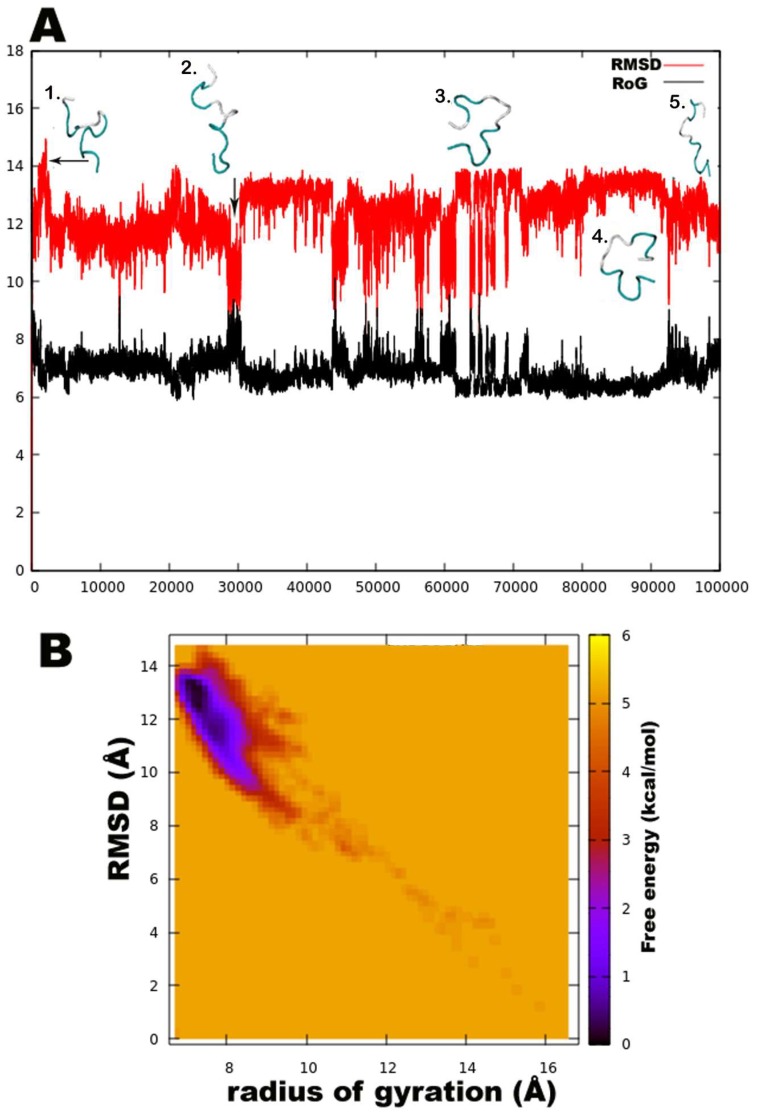
(**A**). The root-mean-square-deviation (RMSD in red color) and radius of gyration (RoG in black) with corresponding three-dimensional structures of trikoningin KA V, (**B**). Free energy landscape as a function of RMSD and RoG shows energetically favoured conformations with RMSD between 12–14 Å and RoG value less than 8 Å.

Unexpectedly, a strong preference was found for the left-handed helix region of Ф-ψ plots during this simulation, specifically for residues in the central region flanked by Gln6, Aib7, Aib8, Aib9, Aib12, Val14, Aib15, and Ile16. Except for Aib1 and Aib4, all other Aib residues show free energy minimum in the left-handed helix region. Most standard (proteinogenic) amino acid residues, Gly2, Ala3, Ser10, Leu11, Gln17, and Gln18, display an energy minimum in the right-handed α-helix region. Ile5, Ser10, Leu11, Pro13, and Ile16 also show preference for a poly-proline II region. This behaviour of Leu and Ile to occupy β-space on the Ramachandran plot is expected due to β-branching of their side-chains. It is known that due to heavy side chains, they show lesser propensity to exist in a helix and, therefore, prefer formation of β-strands. The presence of three consecutive Aib residues in positions 7, 8, and 9 of the peptide chain seems to drive its conformation towards a left-handed helix, while the rest shows clear preference for right-handedness. This resulted in an overall unwinding of the helix, which never seems to form a continuous spiral shape. Further experiments with higher sampling power are required to confirm these results. The calculation of root-mean-square-deviation (RMSD) values based on the coordinates of peptide backbone atoms C, CA, and N for each frame with respect to the average structure has been provided. A similar result was obtained for radius of gyration (RoG) values, which is the root-mean-square-distance of peptide components from their center of mass calculated for each frame. The preliminary investigation revealed that the overall conformation (obtained from the trajectory with RMSD value between 12 to 14Å, denoted by structures 3 and 4) resembles a hairpin with turn structures that never assumes a spiral shape (Figure 2A). Structures 2 and 5 with an almost linear structure show lower RMSD values than 12 Å, which is not energetically favoured. The free-energy landscape as a function of RMSD and radius of gyration is shown in Figure 2B, which clearly indicates that structures with RoG value of less than 8 Å and RMSD values between 12–14 Å are energetically favoured. When compared with the hydrogen bonding pattern within the backbone, mostly i+3→i H-bonds were found that denote 3_10_ helix probably in left-handed conformation as indicated by Ф-ψ plots (Table 3). Ile5→Gly2, Aib8→Ile5, Aib9→Gln6, Ser10→Aib7, Ile16→Pro13, and Gln17→Val14 are examples of left-handed 3_10_ helix bonds while Gln6→Ala3, Leuol19→Ile16, Leu11→Aib8 are examples of right-handed 3_10_ helix. Few γ-turn populations are also seen by Aib7→Ile5, Aib12→Ser10, and Gln17→Aib15 as energetically stable. This means that the highly bent structure resembling a β-hairpin with the N- and C-terminals in close proximity to each other is energetically favoured in comparison to a linear backbone.

### 3.3. Inhibitory Effects of Peptaibol Extracts Towards Bacteria, Yeasts, and Filamentous Fungi

*Micrococcus luteus* and *S. aureus* proved to be sensitive to both peptaibol extracts (Table 4), *E. coli* was more resistant, while *P. aeruginosa* showed higher sensitivity to the extracts from *T. koningiopsis* than to those from *T. gamsii*. *M. luteus* and *S. aureus* are Gram-positive bacteria, while *E. coli* and *P. aeruginosa* are Gram-negative ones, thus their sensitivity showed correlation with the type of their cell wall. Studies on the bioactivity of paracelsins [60] or alamethicin [61] showed similar results, i.e., that Gram-positive bacteria proved to be more sensitive to peptaibols. Testing the peptaibols trichorzianine A1 and B1 on Gram-positive and Gram-negative bacteria also revealed similar results, furthermore, synergistic effect could also be detected between peptaibols and different cell membrane-affecting (MACs) and cell wall-degrading enzymes (CWDEs) [62,63]. In a study of Lorito et al. [64], the inhibition of β-D-glucan synthase was reported as a specific effect of peptaibol antibiotics. On the other hand, in the study of Cutler et al. [65], the purified peptaibol identified as trikoningin KA V (also known as koningin A) seemed to be inactive against both Gram-positive and Gram-negative bacteria. No inhibition zones could be observed in the case of yeasts (Table 4), though in another study, minimum inhibition could be observed after the treatment of trichokonins produced by *T. koningii* on *S. cerevisiae* CGMCC2.395 and *C. albicans* CGMCC2.538 [66]. Complete inhibition could not be observed among the fast-growing fungi (Table 5), though. Interestingly, the producer *T. koningiopsis* and *T. gamsii* strains seemed to be more sensitive to their own peptaibol extracts than *T. aggressivum* f. *europaeum* and *T. pleuroti*, known as the causal agents of green mould disease occurring in mushroom cultivation (Table 5). The latter two species are also known to produce peptaibols [67], but their peptaibiomes are entirely different from those of *T. koningiopsis* SZMC 12500 and *T. gamsii* SZMC 1656, they produce 18-residue hypomurocin-like peptaibols and tripleurins, respectively.

The *F. solani* species complex member appeared to be more sensitive to the peptaibol extract of *T. gamsii* than to the one of *T. koningiopsis*. Inhibition could also be detected in the case of *A. alternata*, *R. solani* and *P. cucurbitacearum*, all growing very slowly (8 days, 12 days, and 11 days, respectively, till they reach the MeOH hole on the plates), and interestingly, the mycelial growth of these filamentous fungi stopped where the peptaibols were added into the holes and could not reach the edge of the plates (Table 5).

## 4. Conclusions

In this study, the peptaibiome composition of *T. koningiopsis* and *T. gamsii* was identified by HPLC-ESI-MS measurements, which revealed a total of 30 peptaibol sequences. A structurally close compound, trikoningin KA V, was selected from the literature for structural elucidation, which revealed fluctuating right- and left-handed helical conformations. The examination of their antibiotic activity against a broad spectrum of different microorganisms showed that Gram-positive bacteria were strongly inhibited, while Gram-negative bacteria seemed to be less sensitive to the peptaibol extracts tested. Inhibitory effects of the studied peptaibol extracts could not be observed on yeasts, while filamentous fungi showed considerable sensitivity.

## Figures and Tables

**Table 1:** Sequences of the newly identified peptaibol compounds produced by *T. gamsii* SZMC 1656 and their similarities to known peptaibols available in the “Peptaibiotics Database”.

Peptide	M	[M+Na]^+^	[M+2Na]^2+^	b_12_	y_7_	rt-GK (min)	Area size	R1	R2	R3	R4	R5	R6	R7	R8	R9	R10	R11	R12	R13	R14	R15	R16	R17	R18	R19	Compound identical or positionally isomeric with	Reference
Pept-Ia	1861.1	1884.1	953.55	1108.5	754.4	50.504	0.28%	AcAib	*Gly*	*Ala*	Aib	*Vxx*	Gln	Aib	Aib	Aib	Ser	Lxx	Aib	Pro	Vxx	Aib	*Vxx*	Gln	Gln	Lxxol	**New:** Trikoningin KA V: [Ile]^5^→[*Vxx*]^5^ and [Ile]^16^→[*Vxx*]^16^	[39,40]
																											**New:** Tricholongin LBII, LBIV: [Phe]^3^→[*Ala*]^3^ and [Aib]^5^→[*Vxx*]^5^	[41]
																											**New:** Tricholongin BII (LBII): [Phe]^3^→[*Ala*]^3^ and [Aib]^5^→[*Vxx*]^5^	[44]
Pept-Ib	1876.2	1899.2	961.1	1108.5	768.4	51.073	1.51%	AcAib	Gly	Ala	Aib	*Vxx*	Gln	Aib	Aib	Aib	Ser	Lxx	Aib	Pro	Vxx	Aib	Lxx	Gln	Gln	Lxxol	**New:** Trikoningin KA V: [Ile]^5^→[*Vxx*]^5^ (Positional isomer of Pept-IIb;	[39,40]
																											Pept-IIIb; Pept-IVb)	
Pept-IIa	1876.1	1899.1	961.05	1122.5	754.4	51.175	1.66%	AcAib	Gly	*Ala*	Aib	*Lxx*	Gln	Aib	Aib	Aib	Ser	Lxx	Aib	Pro	Vxx	Aib	*Vxx*	Gln	Gln	Lxxol	**New:** Trikoningin KA V: [Ile]^16^→[*Vxx*]^16^ (Positional isomer of Pept-IIIa;	[39,40]
																											Pept-IVa; Pept-Va; Pept-VIa; Pept-VIIIa)	
																											**New:** Tricholongin LBII, LBIV: [Phe]^3^→[*Ala*]^3^ and [Aib]^5^→[*Lxx*]^5^	[41]
																											**New:** Tricholongin BII: [Phe]^3^→[*Ala*]^3^ and [Aib]^5^→[*Lxx*]^5^	[44]
																											**New:** Trichostrigocin TSG-A, TSG-B: [Ala]^2^→[*Gly*]^2^ and [Aib]^3^→[Ala]^3^	[41]
Pept-IIb	1875.2	1898.2	960.6	1108.5	768.4	52.316	1.99%	AcAib	Gly	Ala	Aib	*Vxx*	Gln	Aib	Aib	Aib	Ser	Lxx	Aib	Pro	Vxx	Aib	Lxx	Gln	Gln	Lxxol	**New:** (Positional isomer of Pept-Ib; Pept-IIIb; Pept-IVb)	→ Pept-Ib
Pept-IIIa	1876.2	1899.2	961.1	1122.5	754.4	53.005	0.56%	AcAib	*Gly*	*Ala*	Aib	*Lxx*	Gln	Aib	Aib	Aib	Ser	Lxx	Aib	Pro	Vxx	Aib	*Vxx*	Gln	Gln	Lxxol	**New:** (Positional isomer of Pept-IIa; Pept-IVa; Pept-Va; Pept-VIa; Pept-VIIIa)	→ Pept-IIa
Pept-IIIb	1875.3	1898.3	960.65	1108.5	768.4	53.292	2.96%	AcAib	Gly	Ala	Aib	*Vxx*	Gln	Aib	Aib	Aib	Ser	Lxx	Aib	Pro	Vxx	Aib	Lxx	Gln	Gln	Lxxol	**New:** (Positional isomer of Pept-Ib; Pept-IIb; Pept-IVb)	→ Pept-Ib
Pept-IVa	1875.2	1898.2	960.6	1122.5	754.4	53.555	3.70%	AcAib	*Gly*	*Ala*	Aib	*Vxx*	Gln	Aib	Aib	Aib	Ser	Lxx	Aib	Pro	Vxx	Aib	*Vxx*	Gln	Gln	Lxxol	**New:** (Positional isomer of Pept-IIa; Pept-IIIa; Pept-Va; Pept-VIa; Pept-VIIIa)	→ Pept-IIa
Pept-IVb	1875.2	1898.2	960.6	1108.5	768.4	53.996	9.21%	AcAib	Gly	Ala	Aib	*Vxx*	Gln	Aib	Aib	Aib	Ser	Lxx	Aib	Pro	Vxx	Aib	Lxx	Gln	Gln	Lxxol	**New:** (Positional isomer of Pept-Ib; Pept-IIb; Pept-IIIb)	→ Pept-Ib
Pept-Va	1876.2	1899.2	961.1	1122.5	754.4	54.776	4.49%	AcAib	*Gly*	*Ala*	Aib	*Lxx*	Gln	Aib	Aib	Aib	Ser	Lxx	Aib	Pro	Vxx	Aib	*Vxx*	Gln	Gln	Lxxol	**New:** (Positional isomer of Pept-IIa; Pept-IIIa; Pept-IVa; Pept-VIa; Pept-VIIIa)	→ Pept-IIa
Pept-Vb	1890.2	1913.2	968.1	1122.5	768.4	55.313	5.41%	AcAib	Gly	Ala	Aib	Lxx	Gln	Aib	Aib	Aib	Ser	Lxx	Aib	Pro	Vxx	Aib	Lxx	Gln	Gln	Lxxol	Trikoningin KA V (Positional isomer of Pept-VIb; Pept-VII; Pept-VIIIb)	[39,40]
Pept-VIa	1876.2	1899.2	961.1	1122.5	754.4	55.617	0.84%	AcAib	*Gly*	*Ala*	Aib	*Lxx*	Gln	Aib	Aib	Aib	Ser	Lxx	Aib	Pro	Vxx	Aib	*Vxx*	Gln	Gln	Lxxol	**New:** (Positional isomer of Pept-IIa; Pept-IIIa; Pept-IVa; Pept-Va; Pept-VIIIa)	→ Pept-IIa
Pept-VIb	1890.2	1913.2	968.1	1122.5	768.4	55.798	23.32%	AcAib	Gly	Ala	Aib	Lxx	Gln	Aib	Aib	Aib	Ser	Lxx	Aib	Pro	Vxx	Aib	Lxx	Gln	Gln	Lxxol	(Positional isomer of Pept-Vb; Pept-VII; Pept-VIIIb)	→ Pept-Vb
Pept-VII	1889.3	1912.3	967.65	1122.5	768.4	56.403	4.16%	AcAib	Gly	Ala	Aib	Lxx	Gln	Aib	Aib	Aib	Ser	Lxx	Aib	Pro	Vxx	Aib	Lxx	Gln	Gln	Lxxol	(Positional isomer of Pept-Vb; Pept-VIb; Pept-VIIIb)	→ Pept-Vb
Pept-VIIIa	1874.2	1897.2	960.1	1122.5	754.4	56.806	0.49%	AcAib	*Gly*	*Ala*	Aib	*Lxx*	Gln	Aib	Aib	Aib	Ser	Lxx	Aib	Pro	Vxx	Aib	*Vxx*	Gln	Gln	Lxxol	**New:** (Positional isomer of Pept-IIa; Pept-IIIa; Pept-IVa; Pept-Va; Pept-VIa)	→ Pept-IIa
Pept-VIIIb	1890.2	1913.2	968.1	1122.5	768.4	56.826	27.99%	AcAib	Gly	Ala	Aib	Lxx	Gln	Aib	Aib	Aib	Ser	Lxx	Aib	Pro	Vxx	Aib	Lxx	Gln	Gln	Lxxol	(Positional isomer of Pept-Vb; Pept-VIb; Pept-VII)	→ Pept-Vb
Pept-IX	1874.5	1897.5	960.25	1106.5	768.4	57.554	5.92%	AcAib	Gly	*Ala*	Aib	*Lxx*	Gln	Aib	Aib	Aib	Ala	Lxx	Aib	Pro	Vxx	Aib	*Lxx*	Gln	Gln	Lxxol	**New:** Tricholongin LBIII: [Phe]^3^→[*Ala*]^3^, [Aib]^5^→[*Lxx*]^5^ and [Lxx]^16^→[*Vxx*]^16^	[41]
Pept-X	1904.2	1927.2	975.1	1136.4				AcAib	*Ala*	*Ala*	Aib	*Lxx*	Gln	Aib	Aib	Aib	Ser	Lxx	Aib	Pro	Vxx	Aib	*Lxx*	Gln	Gln	*Lxxol*	**New:** Trikoningin KA V: Gly]^2^→[*Ala*]^2^ (Positional isomer of Pept-XI; Pept-XII)	[39,40]
																											**New:** Trichostrigocin TSG-A, TSG-B: [Aib]^3^→[*Ala*]^3^ and [Vxx]^16^→[*Lxx*]^16^	[41]
																											**New:** Trichorzianin TA IIIb, TA IIIb: [Aib]^5^→[*Lxx*]^5^ and [Trpol]^19^→[*Lxxol*]^19^	[45]
																											**New:** Trichorzianin TA VIb: [Aib]^5^→[*Lxx*]^5^ and [Pheol]^19^→[*Lxxol*]^19^	[45]
																											**New:** Trichorzianin TA IVb: [Iva]^5^→[L*xx*]^5^ and [Trpol]^19^→[*Lxxol*]^19^	[45]
																											**New:** Trichorzianin TA VII: [Iva]^5^→[L*xx*]^5^ and [Pheol]^19^→[*Lxxol*]^19^	[45]
																											**New:** Trichorzianin TAP 14a: [Aib]^5^→[*Lxx*]^5^ and [Trpol]^19^→[*Lxxol*]^19^	[46]
																											**New:** Trichorzianin TB IIIc: [Aib]^5^→[*Lxx*]^5^ and [Trpol]^19^→[*Lxxol*]^19^	[47]
																											**New:** Trichorzianin TB IVb: [Iva]^5^→[L*xx*]^5^ and [Trpol]^19^→[*Lxxol*]^19^	[47]
																											**New:** Trichorzianin TB VIb: [Aib]^5^→[*Lxx*]^5^ and [Pheol]^19^→[*Lxxol*]^19^	[47]
																											**New:** Trichorzianin TB VII: [Iva]^5^→[L*xx*]^5^ and [Pheol]^19^→[*Lxxol*]^19^	[47]
Pept-XI	1903.3	1926.3	974.65	1136.5	768.4	58.602	4.82%	AcAib	*Ala*	*Ala*	Aib	*Lxx*	Gln	Aib	Aib	Aib	Ser	Lxx	Aib	Pro	Vxx	Aib	*Lxx*	Gln	Gln	*Lxxol*	**New:** (Positional isomer of Pept-X; Pept-XII)	→ Pept-X
Pept-XII	1904.2	1927.2	975.1	1136.6	768.4	59.264	0.69%	AcAib	*Ala*	*Ala*	Aib	*Lxx*	Gln	Aib	Aib	Aib	Ser	Lxx	Aib	Pro	Vxx	Aib	*Lxx*	Gln	Gln	*Lxxol*	**New:** (Positional isomer of Pept-X; Pept-X)	→ Pept-X

Variable residues are underlined in the table header, minor sequence variants are underlined in the sequences. Amino acid exchanges in new sequences are italicised.

**Table 2:** Sequences of the newly identified peptaibol compounds produced by *T. koningiopsis* SZMC 12500 and their similarities to known peptaibols available in the “Peptaibiotics Database”.

Peptide	M	[M+Na]^+^	[M+2Na]^2+^	b_12_	y_7_	rt-GK (min)	Area size	R1	R2	R3	R4	R5	R6	R7	R8	R9	R10	R11	R12	R13	R14	R15	R16	R17	R18	R19	Compound identical or positionally isomeric with	Reference
Koningiopsin Ia	1875.5	1898.5	960.75	1121.9	754.5	53.121	0.91%	AcAib	Ala	Ala	Aib	Vxx	Gln	Aib	Aib	Aib	Ser	Lxx	Aib	Pro	Vxx	Aib	Vxx	Gln	Gln	*Lxxol*	**New:** Trichorzianin TAP-14b: [Pheol]^19^→[*Lxxol*]^19^	[46]
Koningiopsin Ib	1889.5	1912.5	967.75	1121.5	768.5	54.267	5.96%	AcAib	Ala	Ala	Aib	Vxx	Gln	Aib	Aib	Aib	Ser	Lxx	Aib	Pro	Vxx	Aib	Lxx	Gln	Gln	*Lxxol*	**New:** Trichorzianin TA IVb: [Trpol]^19^→[*Lxxol*]^19^	[45]
																											**New:** Trichorzianin TA VII: [Pheol]^19^→[*Lxxol*]^19^	[45]
Koningiopsin IIa	1890.5	1913.5	968.25	1136.7	754.5	54.69	8.13%	AcAib	Ala	*Ala*	Aib	Aib	Gln	Aib	Aib	*Lxx*	Ser	Lxx	Aib	Pro	Vxx	Aib	Vxx	Gln	Gln	*Lxxol*	**New:** Trichorzianin TA IVb: [Aib]^9^→[*Lxx*]^9^ and [Trpol]^19^→[*Lxxol*]^19^	[45]
																											**New:** Trichostrigocin TSG-A, TSG-B:[Aib]^3^→[*Ala*]^3^ and [Aib]^9^→[*Lxx*]^9^	[41]
Koningiopsin IIb	1889.6	1912.6	967.8	1120.8	768.6	55.513	2.88%	AcAib	Ala	Ala	Aib	Aib	Gln	Aib	Aib	Aib	*Vxx*	Lxx	Aib	Pro	Vxx	Aib	Lxx	Gln	Gln	*Lxxol*	**New:** Trichorzianin TA IIIb, TA IIIc: [Ser]^10^→[*Vxx*]^10^ and	[45]
																											[Trpol]^19^→[*Lxxol*]^19^	
																											**New:** Trichorzianin TA VIb: [Ser]^10^→[*Vxx*]^10^ and [Pheol]^19^→[*Lxxol*]^19^	[45]
																											**New:** Trichorzianin TAP-14a: [Ser]^10^→[*Vxx*]^10^ and [Pheol]^19^→[*Lxxol*]^19^	[46]
Koningiopsin IIIa	1891.6	1914.6	968.8	1122.7	755.6	55.593	0.97%	AcAib	Ala	*Ala*	Aib	*Ala*	Gln	Aib	Aib	*Lxx*	Ser	Lxx	Aib	Pro	Vxx	Aib	*Vxx*	Gln	*Glu*	*Lxxol*	**New:** Trichorzianin TAP-14b: [Aib]^5^→[*Ala*]^5^, [Aib]^9^→[*Lxx*]^9^,	[46]
																											[Gln]^18^→[*Glu*]^18^ and [Pheol]^19^→[*Lxxol*]^19^	
																											**New:** Trichorzianin TB IIIc, TB IVb: [Aib]^5^→[*Ala*]^5^, [Aib]^9^→[*Lxx*]^9^, [Ile]^16^→[*Vxx*]^16^ and [Trpol]^19^→[*Lxxol*]^19^	[47]
																											**New:** Trichorzianin TB VIb, TB VII: [Aib]^5^→[*Ala*]^5^, [Aib]^9^→[*Lxx*]^9^, [Ile]^16^→[*Vxx*]^16^ and [Trpol]^19^→[*Lxxol*]^19^	[47]
																											**New:** Trichostrigocin TSG-A, TSG-B: [Aib]^3^→[*Ala*]^3^, [Ala]^5^→[*Lxx*]^5^, [Aib]^9^→[*Lxx*]^9^ and [Gln]^18^→[*Glu*]^18^	[41]
Koningiopsin IIIb	1873.6	1896.6	959.8	1120.8	754.5	55.936	2.03%	AcAib	Ala	Ala	Aib	Aib	Gln	Aib	Aib	Aib	*Vxx*	Lxx	Aib	Pro	Vxx	Aib	Vxx	Gln	Gln	*Lxxol*	**New:** Trichorzianin TAP-14b: [Ser]^10^→[*Vxx*]^10^ and [Pheol]^19^→[*Lxxol*]^19^	[46]
Koningiopsin IV	1903.6	1926.6	974.8	1136.7	768.6	56.317	12.27%	AcAib	Ala	Ala	Aib	Aib	Gln	Aib	Aib	Lxx	Ser	Lxx	Aib	Pro	Vxx	Aib	Lxx	Gln	Gln	*Lxxol*	**New:** Trichorzianin TA IIIb, TA IIIc: [Aib]^9^→[*Lxx*]^9^ and	[45]
																											[Trpol]^19^→[*Lxxol*]^19^ (Positional isomer of Koningiopsin Va)	
																											**New:** Trichorzianin TA VIb: [Aib]^9^→[*Lxx*]^9^ and [Pheol]^19^→[*Lxxol*]^19^	[45]
																											**New:** Trichorzianin TAP-14a: [Aib]^9^→[*Lxx*]^9^ and [Pheol]^19^→[*Lxxol*]^19^	[46]
Koningiopsin Va	1903.6	1926.6	974.8	1136.8	768.6	56.739	47.94%	AcAib	Ala	Ala	Aib	Aib	Gln	Aib	Aib	*Lxx*	Ser	Lxx	Aib	Pro	Vxx	Aib	Lxx	Gln	Gln	*Lxxol*	**New:** (Positional isomer of Koningiopsin IV)	→Koningiopsin IV
Koningiopsin Vb	1905.6	1928.6	975.8	1136.8	769.6	57.463	5.95%	AcAib	Ala	Ala	Aib	Aib	Gln	Aib	Aib	*Lxx*	Ser	Lxx	Aib	Pro	Vxx	Aib	Lxx	Gln	Glu	*Lxxol*	**New:** Trichorzianin TB IIIc: [Aib]^9^→[*Lxx*]^9^, and [Trpol]^19^→[*Lxxol*]^19^	[47]
																											**New:** Trichorzianin TBVIb: [Aib]^9^→[*Lxx*]^9^, and [Pheol]^19^→[*Lxxol*]^19^	[47]
Koningiopsin VIa	1888.6	1911.6	967.3	1120.7	768.6	57.825	11.54%	AcAib	Ala	Ala	Aib	Aib	Gln	Aib	Aib	*Lxx*	*Ala*	Lxx	Aib	Pro	Vxx	Aib	Lxx	Gln	Gln	*Lxxol*	**New:** Trichorzianin TA IIIb, TA IIIc: [Aib]^9^→[*Lxx*]^9^, [Ser]^10^→[*Ala*]^10^	[45]
																											and [Trpol]^19^→[*Lxxol*]^19^	
																											**New:** Trichorzianin TA VIb: [Aib]^9^→[*Lxx*]^9^, [Ser]^10^→[*Ala*]^10^ and [Pheol]^19^→[*Lxxol*]^19^	[45]
																											**New:** Trichorzianin TAP-14a: [Aib]^9^→[*Lxx*]^9^, [Ser]^10^→[*Ala*]^10^ and [Pheol]^19^→[*Lxxol*]^19^	[46]
Koningiopsin VIb	1888.7	1911.7	967.35	1120.9	769.5	58.448	1.41%	AcAib	Ala	Ala	Aib	Aib	Gln	Aib	Aib	*Lxx*	*Ala*	Lxx	Aib	Pro	Vxx	Aib	Lxx	Gln	Glu	*Lxxol*	**New:** Trichorzianin TB IIIc: [Aib]^9^→[*Lxx*]^9^, [Ser]^10^→[*Ala*]^10^ and	[47]
																											[Trpol]^19^→[*Lxxol*]^19^	
																											**New:** Trichorzianin TB VIb: [Aib]^9^→[*Lxx*]^9^, [Ser]^10^→[*Ala*]^10^ and [Pheol]^19^→[*Lxxol*]^19^	[47]

Variable residues are underlined in the table header, minor sequence variants are underlined in the sequences. Amino acid exchanges in new sequences are italicised. Positions R14 and R15 were predicted based on the Comprehensive Peptaibotics Database [14].

**Table 3:** Backbone H-bonds of Trikoningin KA V along with their frequency of occurrence given by fraction, average distance, and angle.

Acceptor	Donor	Fraction	Average Distance
Gly_2	Aib_7	0.2038	2.8926
Gln_6	Aib_9	0.1888	2.8999
Ala_3	Gln_6	0.175	2.8978
Pro_13	Ile_16	0.1729	2.8903
Ile_16	Leu_19	0.1611	2.8976
Aib_8	Leu_11	0.1475	2.8926
Aib_7	Ser_10	0.1211	2.8964
Val_14	Gln_17	0.0917	2.9024
Ile_5	Aib_8	0.0727	2.912
Aib_15	Gln_17	0.0642	2.8104
Aib_15	Gln_18	0.0546	2.9034
Ile_16	Gly_2	0.0457	2.8761
Aib_15	Gln_6	0.0418	2.8816
Ser_10	Aib_12	0.0393	2.846
Ile_5	Aib_7	0.0352	2.8111
Gly_2	Ile_5	0.0344	2.9118
Gln_18	Val_14	0.0316	2.851
Gln_17	Gly_2	0.0303	2.878
AIB_8	AIB_12	0.0303	2.9031

**Table 4:** Bioactivity of concentrated peptaibol extracts (100 mg mL^−1^) from *Trichoderma gamsii* SZMC 1656 and *T. koningiopsis* SZMC 12500 towards bacteria and yeasts.

Tested Microbial Strain	Sensitivity to *T. gamsii* SZMC 1656 Extract	Sensitivity to *T. koningiopsis* SZMC 12500 Extract
*Micrococcus luteus SZMC 0264*	+++++	++++
*Staphylococcus aureus SZMC 0579*	+++++	++++
*Escherichia coli SZMC 0582*	+	−
*Pseudomonas aeruginosa SZMC 0568*	+	++
*Candida boidinii SZMC 0673*	−	−
*Kluyveromyces lactis SZMC 0683*	−	−
*Saccharomyces cerevisiae SZMC 0425*	−	−
*Schizosaccharomyces pombe SZMC 0142*	−	−

−, absence of inhibition; diameter of inhibition zone: +, 5–7 mm; ++, 7–9 mm, +++, 9–11 mm, ++++, 11–13 mm diameter, +++++, 13–15 mm.

**Table 5:** Bioactivity of concentrated peptaibol extracts (100 mg mL^−1^) and their two-fold serial dilutions from *Trichoderma gamsii* SZMC 1656 and *T. koningiopsis* SZMC 12500 towards cultures of filamentous fungi.

Tested Filamentous Fungal Strain	Sensitivity to *T. gamsii* SZMC 1656 Extract	Sensitivity to *T. koningiopsis* SZMC 12500 Extract
*Alternaria alternata* SZMC 16085	+++++ *	+++ *
*Fusarium solani* species complex SZMC 11467	++	+
*Rhizoctonia solani* SZMC 6252J	++ *	+++ *
*Phoma cucurbitacearum* SZMC 16088	++ *	++++ *
*T. aggressivum* f. *europaeum* SZMC 1811	++	+
*T. pleuroti* SZMC 12454	++	+
*T. gamsii* SZMC 1656	+++	+++
*T. koningiopsis* SZMC 12500	+++	+++

inhibition of mycelial growth at dilution steps: +, 1st–2nd; ++, 3rd–4th; +++, 5th; ++++, 6th; +++++, 7 th. *, mycelial growth was completely stopped.

## Supplementary Materials

The following are available online at http://www.mdpi.com/2076-2607/6/3/85/s1, Supplementary Data 1: Detailed description for residue parameterization, Figure S1: Extracted ion chromatograms (EIC) resulting from full scan measurements of crude extracts from *T. gamsii* SZMC 1656, Figure S2: Extracted ion chromatograms (EIC) resulting from full scan measurements of crude extracts prepared from *T. koningiopsis* SZMC 12500, Figure S3: Typical b ion series of described peptaibols ranged from b_12_ at *m/z* 1106, Figure S4: Typical b ion series of described peptaibols ranging from b_12_ at *m/z* 1120, Figure S5: Typical MS^2^ spectra of y-ions 754.5 (A), 755.5 (B), 768.5 (C) and 769.5 (D) resulting from the full scan measurements of crude peptaibol extracts, Table S1: Diagnostic fragment ions of peptaibols detected with the full scan MS measurement of peptaibol extracts from plate cultures of *T. gamsii* SZMC 1656, Table S2: Diagnostic fragment ions of peptaibols detected with the full scan MS measurement of peptaibol extracts from plate cultures of *T. koningiopsis* SZMC 12500, Table S3: Diagnostic fragment ions of acylium ion (y_7_) detected with MS^2^ measurements of peptaibol extracts from plate cultures of *T. gamsii* SZMC 1656, Table S4: Diagnostic fragment ions of acylium ion (y_7_) detected with MS^2^ measurements of peptaibol extracts from plate cultures of *T. koningiopsis* SZMC 12500.
