# Supplementary material for: New 19-Residue Peptaibols from Trichoderma Clade Viride
Source: Microorganisms. 2018 Aug 12;6(3):85. doi: 10.3390/microorganisms6030085 (PMC6165201; doi:10.3390/microorganisms6030085)
Supplement: Supplementary file 1 [file microorganisms-06-00085-s001.pdf]

Supplementary Data 1. Detailed description for residue parameterization

The non-standard residue, Aib (aminoisobutyric acid) was parameterized as part of one calculation along with 4 standard residues, alanine, serine, glycine and valine. The parameter file, .frcmod, is calculated collectively for all these residues and is provided below. To create their amber libraries in .off format, the respective structures of these residues were loaded using a leap script provided by R.E.D server. The reason for collective parameterization was to be able to compare the charges for standard residues with preexisting libraries of AmberTools16.

Note: The mol2 files contain information of Aib as residue units with free ends that can be readily used in building a peptide sequence. They were parameterized as ACE-AIB-NME units.

Using leap,

```
loadAmberParams frcmod.known                    #parameter file for all 5 residues
AIB = loadmol3 m3-c1_f2.mol2                 #from R.E.D charge calculation results

saveoff AIB AIB.off                                         #saving amber libraries
```

The .mol2 file for AIB

```
@<TRIPOS>MOLECULE
F08
    13    12     1     0     1
SMALL
USER_CHARGES
@<TRIPOS>ATOM
   1 N1     1.179404   0.787225  -0.289608 N     1    F08 -0.4552  0.0000 ****
   2 H7     1.401639   1.218298  -1.161329 H     1    F08  0.2870  0.0000 ****
   3 C1    -0.246026   0.712307  -0.009072 CT    1    F08  0.1590  0.0000 ****
   4 C2    -0.513722   0.943419   1.490893 CT    1    F08 -0.1445  0.0000 ****
   5 C3    -0.874452   1.859278  -0.822371 CT    1    F08 -0.1445  0.0000 ****
   6 H8    -0.683793   1.725172  -1.881967 HC    1    F08  0.0464  0.0000 ****
   7 H9    -1.944751   1.917349  -0.681560 HC    1    F08  0.0464  0.0000 ****
   8 H10   -0.438130   2.800958  -0.509348 HC    1    F08  0.0464  0.0000 ****
   9 C6    -0.815324  -0.626254  -0.549026 C     1    F08  0.5633  0.0000 ****
  10 O     -0.117607  -1.349894  -1.206062 O     1    F08 -0.5435  0.0000 ****
  11 H1    -1.529198   1.266287   1.670775 HC    1    F08  0.0464  0.0000 ****
  12 H2     0.148540   1.726800   1.836879 HC    1    F08  0.0464  0.0000 ****
  13 H     -0.336510   0.060129   2.090571 HC    1    F08  0.0464  0.0000 ****
@<TRIPOS>BOND
     1     1     2 1
     2     1     3 1
     3     3     4 1
     4     3     5 1
     5     3     9 1
     6     4    11 1
     7     4    12 1
     8     4    13 1
     9     5     6 1
    10     5     7 1
    11     5     8 1
    12     9    10 1
@<TRIPOS>SUBSTRUCTURE
      1 F08              1 ****               0 ****  ****
@<TRIPOS>HEADTAIL
N1 1
```

```
C6 1
@<TRIPOS>RESIDUECONNECT
1 N1 C6 0 0 0 0
```

The parameter file used for AIB residue

```
FRCMOD file generated by PyRED version SEP-2015 - q4md-forcefieldtools.org
MASS      mass               pol            Source
C         12.010             0.616          taken from parm10.dat
CT        12.010             0.878          taken from parm10.dat
CX        12.010             0.360          taken from parm10.dat
H          1.008             0.161          taken from parm10.dat
H1         1.008             0.135          taken from parm10.dat
HC         1.008             0.135          taken from parm10.dat
HO         1.008             0.135          taken from parm10.dat
N         14.010             0.530          taken from parm10.dat
O         16.000             0.434          taken from parm10.dat
OH        16.000             0.465          taken from parm10.dat

BOND   K(kcal.mol-1.ang-2)  Dist0(ang)     Source
C -CT     315.0              1.522          adapted from parm10.dat 317.0
C -CX     315.0              1.522          adapted from parm10.dat 317.0
C -N      490.0              1.335          taken from parm10.dat
C -O      570.0              1.229          taken from parm10.dat
CT-CT     310.0              1.526          taken from parm10.dat
CT-CX     310.0              1.526          taken from parm10.dat
CT-H1     340.0              1.090          taken from parm10.dat
CT-HC     340.0              1.090          taken from parm10.dat
CT-N      335.0              1.449          adapted from parm10.dat 337.0
CT-OH     320.0              1.410          taken from parm10.dat
CX-H1     340.0              1.090          taken from parm10.dat
CX-N      335.0              1.449          adapted from parm10.dat 337.0
H -N      435.0              1.010          adapted from parm10.dat 434.0
HO-OH     555.0              0.960          adapted from parm10.dat 553.0

ANGLE  K(kcal.mol-1.rad-2)  Theta0(deg)    Source
CT-C -N        70.0          116.60         taken from parm10.dat
CT-C -O        80.0          120.40         taken from parm10.dat
CX-C -N        70.0          116.60         taken from parm10.dat
CX-C -O        80.0          120.40         taken from parm10.dat
N -C -O        80.0          122.90         taken from parm10.dat
C -CT-CT       65.0          111.10         adapted from parm10.dat 63.0
C -CT-HC       50.0          109.50         taken from parm10.dat
C -CT-N        65.0          110.10         adapted from parm10.dat 63.0
CT-CT-CT       40.0          109.50         taken from parm10.dat
CT-CT-CX       40.0          109.50         taken from parm10.dat
CT-CT-HC       50.0          109.50         taken from parm10.dat
CT-CT-N        80.0          109.70         taken from parm10.dat
CX-CT-H1       50.0          109.50         taken from parm10.dat
CX-CT-HC       50.0          109.50         taken from parm10.dat
CX-CT-OH       50.0          109.50         taken from parm10.dat
H1-CT-H1       35.0          109.50         taken from parm10.dat
H1-CT-N        50.0          109.50         taken from parm10.dat
H1-CT-OH       50.0          109.50         taken from parm10.dat
HC-CT-HC       35.0          109.50         taken from parm10.dat
C -CX-CT       65.0          111.10         adapted from parm10.dat 63.0
C -CX-H1       50.0          109.50         taken from parm10.dat
C -CX-N        65.0          110.10         adapted from parm10.dat 63.0
CT-CX-H1       50.0          109.50         taken from parm10.dat
CT-CX-N        80.0          109.70         taken from parm10.dat
H1-CX-H1       35.0          109.50         taken from parm10.dat
H1-CX-N        50.0          109.50         taken from parm10.dat
C -N -CT       50.0          121.90         taken from parm10.dat
C -N -CX       50.0          121.90         taken from parm10.dat
C -N -H        50.0          120.00         taken from parm10.dat
CT-N -H        50.0          118.04         taken from parm10.dat
CX-N -H        50.0          118.04         taken from parm10.dat
```

```
CT-OH-HO       55.0          108.50         taken from parm10.dat

DIHEDRAL    Path V(kcal.mol-1.rad-1) Phase(deg.) Period Source
N -C -CT-CT   1    0.00000000e+00        0.0      -4.   taken from parm10.dat
N -C -CT-CT   1    4.00000000e-01        0.0      -3.   taken from parm10.dat
N -C -CT-CT   1    2.00000000e-01        0.0      -2.   taken from parm10.dat
N -C -CT-CT   1    2.00000000e-01        0.0       1.   taken from parm10.dat
N -C -CT-HC   1    0.00000000e+00        0.0       2.   adapted from parm10.dat i.e X-
C-CT-X   0.0/6
N -C -CT-N    1    0.00000000e+00        0.0       2.   adapted from parm10.dat i.e X-
C-CT-X   0.0/6
O -C -CT-CT   1    0.00000000e+00        0.0       2.   adapted from parm10.dat i.e X-
C-CT-X   0.0/6
O -C -CT-HC   1    8.00000000e-01        0.0      -1.   taken from parm10.dat
O -C -CT-HC   1    0.00000000e+00        0.0      -2.   taken from parm10.dat
O -C -CT-HC   1    8.00000000e-02      180.0       3.   taken from parm10.dat
O -C -CT-N    1    0.00000000e+00        0.0       2.   adapted from parm10.dat i.e X-
C-CT-X   0.0/6
N -C -CX-CT   1    0.00000000e+00        0.0      -4.   taken from parm10.dat
N -C -CX-CT   1    4.00000000e-01        0.0      -3.   taken from parm10.dat
N -C -CX-CT   1    2.00000000e-01        0.0      -2.   taken from parm10.dat
N -C -CX-CT   1    2.00000000e-01        0.0       1.   taken from parm10.dat
N -C -CX-H1   1    0.00000000e+00        0.0       2.   adapted from parm10.dat i.e X-
C-CX-X   0.0/6
N -C -CX-N    1    0.00000000e+00        0.0      -4.   taken from parm10.dat
N -C -CX-N    1    5.50000000e-01      180.0      -3.   taken from parm10.dat
N -C -CX-N    1    1.58000000e+00      180.0      -2.   taken from parm10.dat
N -C -CX-N    1    4.50000000e-01      180.0       1.   taken from parm10.dat
O -C -CX-CT   1    0.00000000e+00        0.0       2.   adapted from parm10.dat i.e X-
C-CX-X   0.0/6
O -C -CX-H1   1    8.00000000e-01        0.0      -1.   taken from parm10.dat
O -C -CX-H1   1    0.00000000e+00        0.0      -2.   taken from parm10.dat
O -C -CX-H1   1    8.00000000e-02      180.0       3.   taken from parm10.dat
O -C -CX-N    1    0.00000000e+00        0.0       2.   adapted from parm10.dat i.e X-
C-CX-X   0.0/6
CT-C -N -CT   1    2.50000000e+00      180.0       2.   adapted from parm10.dat i.e X-
C-N-X   10.0/4
CT-C -N -CX   1    2.50000000e+00      180.0       2.   adapted from parm10.dat i.e X-
C-N-X   10.0/4
CT-C -N -H    1    2.50000000e+00      180.0       2.   adapted from parm10.dat i.e X-
C-N-X   10.0/4
CX-C -N -CT   1    2.50000000e+00      180.0       2.   adapted from parm10.dat i.e X-
C-N-X   10.0/4
CX-C -N -H    1    2.50000000e+00      180.0       2.   adapted from parm10.dat i.e X-
C-N-X   10.0/4
O -C -N -CT   1    2.50000000e+00      180.0       2.   adapted from parm10.dat i.e X-
C-N-X   10.0/4
O -C -N -CX   1    2.50000000e+00      180.0       2.   adapted from parm10.dat i.e X-
C-N-X   10.0/4
O -C -N -H    1    2.50000000e+00      180.0      -2.   taken from parm10.dat
O -C -N -H    1    2.00000000e+00        0.0       1.   taken from parm10.dat
C -CT-CT-CT   1    1.55555556e-01        0.0       3.   adapted from parm10.dat i.e X-
CT-CT-X   1.4/9
C -CT-CT-HC   1    1.55555556e-01        0.0       3.   adapted from parm10.dat i.e X-
CT-CT-X   1.4/9
CT-CT-CT-CT   1    1.80000000e-01        0.0      -3.   taken from parm10.dat
CT-CT-CT-CT   1    2.50000000e-01      180.0      -2.   taken from parm10.dat
CT-CT-CT-CT   1    2.00000000e-01      180.0       1.   taken from parm10.dat
CT-CT-CT-HC   1    1.60000000e-01        0.0       3.   taken from parm10.dat
CT-CT-CT-N    1    1.55555556e-01        0.0       3.   adapted from parm10.dat i.e X-
CT-CT-X   1.4/9
CX-CT-CT-HC   1    1.60000000e-01        0.0       3.   taken from parm10.dat
HC-CT-CT-HC   1    1.50000000e-01        0.0       3.   taken from parm10.dat
HC-CT-CT-N    1    1.55555556e-01        0.0       3.   adapted from parm10.dat i.e X-
CT-CT-X   1.4/9
CT-CT-CX-C    1    1.55555556e-01        0.0       3.   adapted from parm10.dat i.e X-
CT-CX-X   1.4/9
```

```
CT-CT-CX-H1   1    1.55555556e-01        0.0        3.   adapted from parm10.dat i.e X-
CT-CX-X   1.4/9
CT-CT-CX-N    1    1.55555556e-01        0.0        3.   adapted from parm10.dat i.e X-
CT-CX-X   1.4/9
H1-CT-CX-C    1    1.55555556e-01        0.0        3.   adapted from parm10.dat i.e X-
CT-CX-X   1.4/9
H1-CT-CX-H1   1    1.55555556e-01        0.0        3.   adapted from parm10.dat i.e X-
CT-CX-X   1.4/9
H1-CT-CX-N    1    1.55555556e-01        0.0        3.   adapted from parm10.dat i.e X-
CT-CX-X   1.4/9
HC-CT-CX-C    1    1.55555556e-01        0.0        3.   adapted from parm10.dat i.e X-
CT-CX-X   1.4/9
HC-CT-CX-H1   1    1.55555556e-01        0.0        3.   adapted from parm10.dat i.e X-
CT-CX-X   1.4/9
HC-CT-CX-N    1    1.55555556e-01        0.0        3.   adapted from parm10.dat i.e X-
CT-CX-X   1.4/9
OH-CT-CX-C    1    1.55555556e-01        0.0        3.   adapted from parm10.dat i.e X-
CT-CX-X   1.4/9
OH-CT-CX-H1   1    0.00000000e+00        0.0       -3.   taken from parm10.dat
OH-CT-CX-H1   1    2.50000000e-01        0.0        1.   taken from parm10.dat
OH-CT-CX-N    1    1.55555556e-01        0.0        3.   adapted from parm10.dat i.e X-
CT-CX-X   1.4/9
C -CT-N -C    1    0.00000000e+00        0.0        2.   adapted from parm10.dat i.e X-
CT-N-X   0.0/6
C -CT-N -H    1    0.00000000e+00        0.0        2.   adapted from parm10.dat i.e X-
CT-N-X   0.0/6
CT-CT-N -C    1    0.00000000e+00        0.0       -4.   taken from parm10.dat
CT-CT-N -C    1    4.00000000e-01        0.0       -3.   taken from parm10.dat
CT-CT-N -C    1    2.00000000e+00        0.0       -2.   taken from parm10.dat
CT-CT-N -C    1    2.00000000e+00        0.0        1.   taken from parm10.dat
CT-CT-N -H    1    0.00000000e+00        0.0        2.   adapted from parm10.dat i.e X-
CT-N-X   0.0/6
H1-CT-N -C    1    0.00000000e+00        0.0        2.   adapted from parm10.dat i.e X-
CT-N-X   0.0/6
H1-CT-N -H    1    0.00000000e+00        0.0        2.   adapted from parm10.dat i.e X-
CT-N-X   0.0/6
CX-CT-OH-HO   1    1.60000000e-01        0.0       -3.   taken from parm10.dat
CX-CT-OH-HO   1    2.50000000e-01        0.0        1.   taken from parm10.dat
H1-CT-OH-HO   1    1.66666667e-01        0.0        3.   adapted from parm10.dat i.e X-
CT-OH-X   0.5/3
C -CX-N -C    1    0.00000000e+00        0.0       -4.   taken from parm10.dat
C -CX-N -C    1    4.20000000e-01        0.0       -3.   taken from parm10.dat
C -CX-N -C    1    2.70000000e-01        0.0       -2.   taken from parm10.dat
C -CX-N -C    1    0.00000000e+00        0.0        1.   taken from parm10.dat
C -CX-N -H    1    0.00000000e+00        0.0        2.   adapted from parm10.dat i.e X-
CX-N-X   0.0/6
CT-CX-N -C    1    0.00000000e+00        0.0       -4.   taken from parm10.dat
CT-CX-N -C    1    4.00000000e-01        0.0       -3.   taken from parm10.dat
CT-CX-N -C    1    2.00000000e+00        0.0       -2.   taken from parm10.dat
CT-CX-N -C    1    2.00000000e+00        0.0        1.   taken from parm10.dat
CT-CX-N -H    1    0.00000000e+00        0.0        2.   adapted from parm10.dat i.e X-
CX-N-X   0.0/6
H1-CX-N -C    1    0.00000000e+00        0.0        2.   adapted from parm10.dat i.e X-
CX-N-X   0.0/6
H1-CX-N -H    1    0.00000000e+00        0.0        2.   adapted from parm10.dat i.e X-
CX-N-X   0.0/6

IMPROPER         V(kcal.mol-1.rad-1) Phase(deg.) Period Source
CT-N -C -O         1.05000000e+01      180.0        2.   adapted from parm10.dat i.e X-
X-C-O
CX-N -C -O         1.05000000e+01      180.0        2.   adapted from parm10.dat i.e X-
X-C-O
C -CT-N -H         1.10000000e+00      180.0        2.   taken from parm10.dat
C -CX-N -H         1.10000000e+00      180.0        2.   taken from parm10.dat

NONBON      R*(ang)   Eps(kcal.mol-1)       Source
C           1.9080    0.08600000            taken from parm10.dat
```

```
CT          1.9080    0.10940000            taken from parm10.dat
CX          1.9080    0.10940000            taken from parm10.dat
H           0.6000    0.01570000            taken from parm10.dat
H1          1.3870    0.01570000            taken from parm10.dat
HC          1.4870    0.01570000            taken from parm10.dat
HO          0.0000    0.00000000            taken from parm10.dat
N           1.8240    0.17000000            taken from parm10.dat
O           1.6612    0.21000000            taken from parm10.dat
OH          1.7210    0.21040000            taken from parm10.dat
```

The .mol2 file for Leuol

```
    0    0    2

This is a remark line
LEO.res
LEO   XYZ  0

  0.0000
   1  DUMM  DU    M        999.000     999.0      -999.0          .00000
   2  DUMM  DU    M        999.000    -999.0       999.0          .00000
   3  DUMM  DU    M       -999.000     999.0       999.0          .00000
   4  N     N     M         -1.110000    0.163000   -0.623000    -0.65514
   5  H     H     E         -1.074000    0.037000   -1.628000     0.33400
   6  CA    CT    M         -0.007000    0.861000    0.018000     0.13835
   7  HA    H1    E         -0.146000    0.721000    1.092000     0.14813
   8  CB    CT    3          1.340000    0.295000   -0.425000    -0.09226
   9  HB2   HC    E          2.127000    0.854000    0.095000     0.04077
  10  HB3   HC    E          1.472000    0.502000   -1.499000     0.04077
  11  CG    CT    3          1.521000   -1.204000   -0.172000     0.16599
  12  HG    HC    E          0.759000   -1.743000   -0.752000    -0.00399
  13  CD1   CT    3          2.895000   -1.651000   -0.671000    -0.21959
  14  HD11  HC    E          3.688000   -1.151000   -0.103000     0.05115
  15  HD12  HC    E          3.035000   -1.408000   -1.730000     0.05115
  16  HD13  HC    E          3.025000   -2.731000   -0.550000     0.05115
  17  CD2   CT    3          1.335000   -1.564000    1.301000    -0.21959
  18  HD21  HC    E          0.318000   -1.367000    1.648000     0.05115
  19  HD22  HC    E          2.029000   -0.987000    1.924000     0.05115
  20  HD23  HC    E          1.545000   -2.626000    1.465000     0.05115
  21  C     CT    M         -0.128000    2.352000   -0.273000     0.21035
  22  H3    H1    E         -1.107000    2.701000    0.082000     0.01873
  23  H1    H1    E         -0.079000    2.520000   -1.362000     0.01873
  24  OAL   OH    S          0.944000    3.007000    0.402000    -0.67748
  25  HAL   HO    E          0.827000    3.964000    0.286000     0.44533


LOOP

IMPROPER
   -M   CA    N    H

DONE
STOP
```

Parameter file for Leuol

```
remark goes here
MASS
N  14.010        0.530               same as n
```

```
H  1.008         0.161               same as hn
CT 12.010        0.878               same as c3
H1 1.008         0.135               same as hc
HC 1.008         0.135               same as hc
OH 16.000        0.465               same as oh
HO 1.008         0.135               same as ho

BOND
N -H   410.20   1.009       same as hn-n
N -CT  330.60   1.460       same as c3-n
CT-H1  337.30   1.092       same as c3-hc
CT-CT  303.10   1.535       same as c3-c3
CT-HC  337.30   1.092       same as c3-hc
CT-OH  314.10   1.426       same as c3-oh
OH-HO  369.60   0.974       same as ho-oh

ANGLE
N -CT-H1   49.800     109.500   same as hc-c3-n
N -CT-CT   65.900     112.130   same as c3-c3-n
H -N -CT   46.000     116.780   same as c3-n -hn
CT-CT-HC   46.400     110.050   same as c3-c3-hc
CT-CT-CT   63.200     110.630   same as c3-c3-c3
CT-CT-H1   46.400     110.050   same as c3-c3-hc
CT-CT-OH   67.700     109.430   same as c3-c3-oh
HC-CT-HC   39.400     108.350   same as hc-c3-hc
CT-OH-HO   47.100     108.160   same as c3-oh-ho
H1-CT-H1   39.400     108.350   same as hc-c3-hc
H1-CT-OH   51.100     109.500   same as hc-c3-oh

DIHE
N -CT-CT-HC   1    0.156          0.000           3.000      same as X -c3-c3-X
N -CT-CT-CT   1    0.156          0.000           3.000      same as X -c3-c3-X
N -CT-CT-H1   1    0.156          0.000           3.000      same as X -c3-c3-X
N -CT-CT-OH   1    0.156          0.000           3.000      same as X -c3-c3-X
H -N -CT-H1   1    0.000          0.000           2.000      same as X -c3-n -X
H -N -CT-CT   1    0.000          0.000           2.000      same as X -c3-n -X
CT-CT-CT-HC   1    0.156          0.000           3.000      same as X -c3-c3-X
CT-CT-CT-CT   1    0.156          0.000           3.000      same as X -c3-c3-X
CT-CT-OH-HO   1    0.167          0.000           3.000      same as X -c3-oh-X
H1-CT-CT-HC   1    0.156          0.000           3.000      same as X -c3-c3-X
H1-CT-CT-CT   1    0.156          0.000           3.000      same as X -c3-c3-X
H1-CT-CT-H1   1    0.156          0.000           3.000      same as X -c3-c3-X
H1-CT-CT-OH   1    0.156          0.000           3.000      same as X -c3-c3-X
CT-CT-CT-OH   1    0.156          0.000           3.000      same as X -c3-c3-X
HC-CT-CT-HC   1    0.156          0.000           3.000      same as X -c3-c3-X
H1-CT-OH-HO   1    0.167          0.000           3.000      same as X -c3-oh-X

IMPROPER

NONBON
  N           1.8240  0.1700             same as n
  H           0.6000  0.0157             same as hn
  CT          1.9080  0.1094             same as c3
  H1          1.4870  0.0157             same as hc
  HC          1.4870  0.0157             same as hc
  OH          1.7210  0.2104             same as oh
  HO          0.0000  0.0000             same as ho
```

Supplementary Table 1. Diagnostic fragment ions of peptaibols detected with the full scan MS measurement of peptaibol extracts from plate cultures of *T. gamsii* SZMC 1656

Peptide	M	[M+Na]$^+$	[M+2Na]$^{2+}$	b_1	b_2	b_3	b_4	b_5	b_6	b_7	b_8	b_9	b_{10}	b_{11}	b_{12}	y_7
Pept-Ia	1861.1	1884.1	953.55	128.0	185.0	256.0	341.0	440.1	n.d.	653.4	738.4	823.5	910.5	1023.8	1108.6	754.5
Pept-Ib	1876.2	1899.2	961.1	128.0	185.0	256.0	341.0	440.1	n.d.	653.4	738.4	823.5	910.5	1023.8	1108.6	768.5
Pept-IIa	1876.1	1899.1	961.05	128.0	185.0	256.0	341.1	454.2	n.d.	667.4	752.5	837.5	924.5	1037.3	1122.6	754.5
Pept-IIb	1875.2	1898.2	960.6	128.0	185.0	256.0	341.0	440.1	n.d.	653.4	738.4	823.5	910.5	1023.8	1108.6	768.5
Pept-IIIa	1876.2	1899.2	961.1	128.0	185.0	256.0	341.1	454.2	n.d.	667.4	752.5	837.5	924.5	1037.3	1122.6	754.5
Pept-IIIb	1875.3	1898.3	960.65	128.0	185.0	256.0	341.0	440.1	n.d.	653.4	738.4	823.5	910.5	1023.8	1108.6	768.5
Pept-IVa	1875.2	1898.2	960.6	128.0	185.0	256.0	341.1	454.2	n.d.	667.4	752.5	837.5	924.5	1037.3	1122.6	754.5
Pept-IVb	1875.2	1898.2	960.6	128.0	185.0	256.0	341.0	440.1	n.d.	653.4	738.4	823.5	910.5	1023.8	1108.6	768.5
Pept-Va	1876.2	1899.2	961.1	128.0	185.0	256.0	341.1	454.2	n.d.	667.4	752.5	837.5	924.5	1037.3	1122.6	754.5
Pept-Vb	1890.2	1913.2	968.1	128.0	185.0	256.0	341.1	454.2	n.d.	667.4	752.5	837.5	924.5	1037.3	1122.6	768.5
Pept-VIa	1876.2	1899.2	961.1	128.0	185.0	256.0	341.1	454.2	n.d.	667.4	752.5	837.5	924.5	1037.3	1122.6	754.5
Pept-VIb	1890.2	1913.2	968.1	128.0	185.0	256.0	341.1	454.2	n.d.	667.4	752.5	837.5	924.5	1037.3	1122.6	768.5
Pept-VII	1889.3	1912.3	967.65	128.0	185.0	256.0	341.1	454.2	n.d.	667.4	752.5	837.5	924.5	1037.3	1122.6	768.5
Pept-VIIIa	1874.2	1897.2	960.1	128.0	185.0	256.0	341.1	454.2	n.d.	667.4	752.5	837.5	924.5	1037.3	1122.6	754.5
Pept-VIIIb	1890.2	1913.2	968.1	128.0	185.0	256.0	341.1	454.2	n.d.	667.4	752.5	837.5	924.5	1037.3	1122.6	768.5
Pept-IX	1874.5	1897.5	960.25	128.0	184.9	256.0	341.3	454.2	n.d.	667.4	752.4	837.5	908.6	1021.7	1106.7	768.5
Pept-X	1904.2	1927.2	975.1	128.0	199.0	270.0	355.0	467.9	n.d.	681.4	766.5	851.5	938.5	1051.0	1136.8	
Pept-XI	1903.3	1926.3	974.65	128.0	199.0	270.0	355.0	467.9	n.d.	681.4	766.5	851.5	938.5	1051.0	1136.8	768.5
Pept-XII	1904.2	1927.2	975.1	128.0	199.0	270.0	355.0	467.9	n.d.	681.4	766.5	851.5	938.5	1051.0	1136.8	768.5

n.d.: not detected

Supplementary Table 2. Diagnostic fragment ions of peptaibols detected with the full scan MS measurement of peptaibol extracts from plate cultures of *T. koningiopsis* SZMC 12500

Peptide	M	[M+Na]$^+$	[M+2Na]$^{2+}$	b_1	b_2	b_3	b_4	b_5	b_6	b_7	b_8	b_9	b_{10}	b_{11}	b_{12}	y_7
Koningiopsin Ia	1875.5	1898.5	960.75	128.0	199.1	270.0	355.1	453.3	n.d.	666.4	751.5	836.6	923.5	1037.6	1122.2	754.5
Koningiopsin Ib	1889.5	1912.5	967.75	128.0	199.1	270.0	355.1	453.3	n.d.	666.4	751.5	836.6	923.5	1037.6	1122.2	768.5
Koningiopsin IIa	1890.5	1913.5	968.25	128.0	199.0	270.0	355.0	440.2	n.d.	653.4	738.4	850.9	938.6	1050.9	1136.8	754.5
Koningiopsin IIb	1889.6	1912.6	967.8	128.1	199.1	270.0	355.1	440.1	n.d.	653.4	738.4	822.5	922.5	1035.8	1120.8	768.5
Koningiopsin IIIa	1891.6	1914.6	968.8	128.1	199.0	270.1	355.1	426.0	n.d.	639.3	724.5	836.4	923.5	1037.1	1122.6	755.5
Koningiopsin IIIb	1873.6	1896.6	959.8	128.1	199.1	270.0	355.1	440.1	n.d.	653.4	738.4	822.5	922.5	1035.8	1120.8	754.5
Koningiopsin IV	1903.6	1926.6	974.8	128.0	199.0	270.0	355.0	440.2	n.d.	653.4	738.4	850.9	938.6	1050.9	1136.8	768.5
Koningiopsin Va	1903.6	1926.6	974.8	128.0	199.0	270.0	355.0	440.2	n.d.	653.4	738.4	850.9	938.6	1050.9	1136.8	768.5
Koningiopsin Vb	1905.6	1928.6	975.8	128.0	199.0	270.0	355.0	440.2	n.d.	653.4	738.4	850.9	938.6	1050.9	1136.8	769.5
Koningiopsin VIa	1888.6	1911.6	967.3	128.1	199.1	270.1	355.0	440.2	n.d.	653.3	738.4	851.6	921.6	1035.8	1120.7	768.5
Koningiopsin VIb	1888.7	1911.7	967.35	128.1	199.1	270.1	355.0	440.2	n.d.	653.3	738.4	851.6	921.6	1035.8	1120.7	769.5

n.d.: not detected

Supplementary Table 3. Diagnostic fragment ions of acylium ion (y_7) detected with MS^2 measurements of peptaibol extracts from plate cultures of *T. gamsii* SZMC 1656

Peptide	y_7	y_7 - H_2O	y_7 - AA (19)	y_7 - AA (19-18)	y_7 - AA (19-17)	y_7 - AA (19-16)
Pept-Ia	754.5	736.5	637.4	509.4	381.4	282.3
Pept-Ib	768.5	750.5	651.4	523.4	395.4	282.4
Pept-IIa	754.5	736.5	637.4	509.4	381.4	282.3
Pept-IIb	768.5	750.5	651.4	523.4	395.4	282.4
Pept-IIIa	754.5	736.5	637.4	509.4	381.4	282.3
Pept-IIIb	768.5	750.5	651.4	523.4	395.4	282.4
Pept-IVa	754.5	736.5	637.4	509.4	381.4	282.3
Pept-IVb	768.5	750.5	651.4	523.4	395.4	282.4
Pept-Va	754.5	736.5	637.4	509.4	381.4	282.3
Pept-Vb	768.5	750.5	651.4	523.4	395.4	282.4
Pept-VIa	754.5	736.5	637.4	509.4	381.4	282.3
Pept-VIb	768.5	750.5	651.4	523.4	395.4	282.4
Pept-VII	768.5	750.5	651.4	523.4	395.4	282.4
Pept-VIIIa	754.5	736.5	637.4	509.4	381.4	282.3
Pept-VIIIb	768.5	750.5	651.4	523.4	395.4	282.4
Pept-IX<br>Pept-X	768.5	750.5	651.4	523.4	395.4	282.4
Pept-XI	768.5	750.5	651.4	523.4	395.4	282.4
Pept-XII	768.5	750.5	651.4	523.4	395.4	282.4

Supplementary Table 4. Diagnostic fragment ions of acylium ion (y_7) detected with MS^2 measurements of peptaibol extracts from plate cultures of *T. koningiopsis* SZMC 12500

Peptide	y_7	y_7 - H_2O	y_7 - AA (19)	y_7 - AA (19-18)	y_7 - AA (19-17)	y_7 - AA (19-16)
Koningiopsin Ia	754.5	736.5	637.4	509.4	381.4	282.3
Koningiopsin Ib	768.5	750.5	651.4	523.4	395.4	282.4
Koningiopsin IIa	754.5	736.5	637.4	509.4	381.4	282.3
Koningiopsin IIb	768.5	750.5	651.4	523.4	395.4	282.4
Koningiopsin IIIa	755.5	737.5	638.4	509.4	381.4	282.3
Koningiopsin IIIb	754.5	736.5	637.4	509.4	381.4	282.3
Koningiopsin IV	768.5	750.5	651.4	523.4	395.4	282.4
Koningiopsin Va	768.5	750.5	651.4	523.4	395.4	282.4
Koningiopsin Vb	769.5	751.5	652.4	523.4	395.3	282.3
Koningiopsin VIa	768.5	750.5	651.4	523.4	395.4	282.4
Koningiopsin VIb	769.5	751.5	652.4	523.4	395.3	282.3

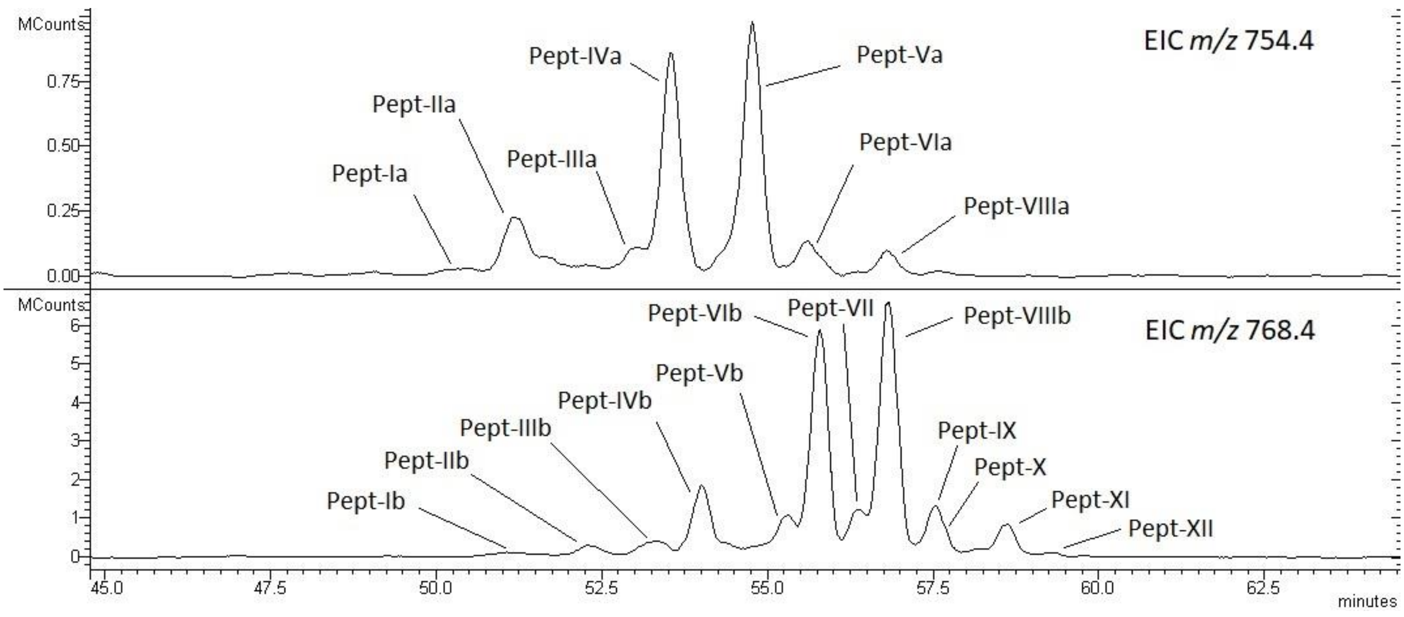

Supplementary Figure 1: Extracted ion chromatograms (EIC) resulting from full scan measurements of crude extracts from *T. gamsii* SZMC 1656. The coeluted components within some peaks were identified based on MS^2 examinations.

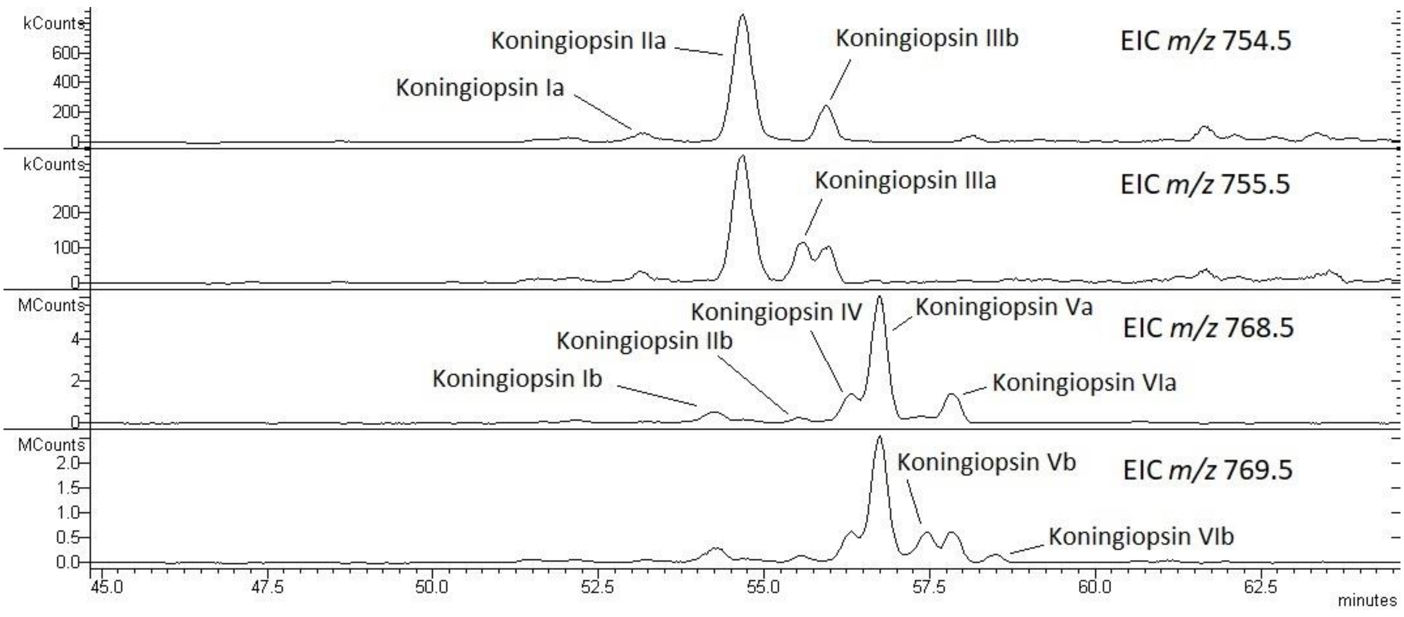

Supplementary Figure 2: Extracted ion chromatograms (EIC) resulting from full scan measurements of crude extracts prepared from *T. koningiopsis* SZMC 12500

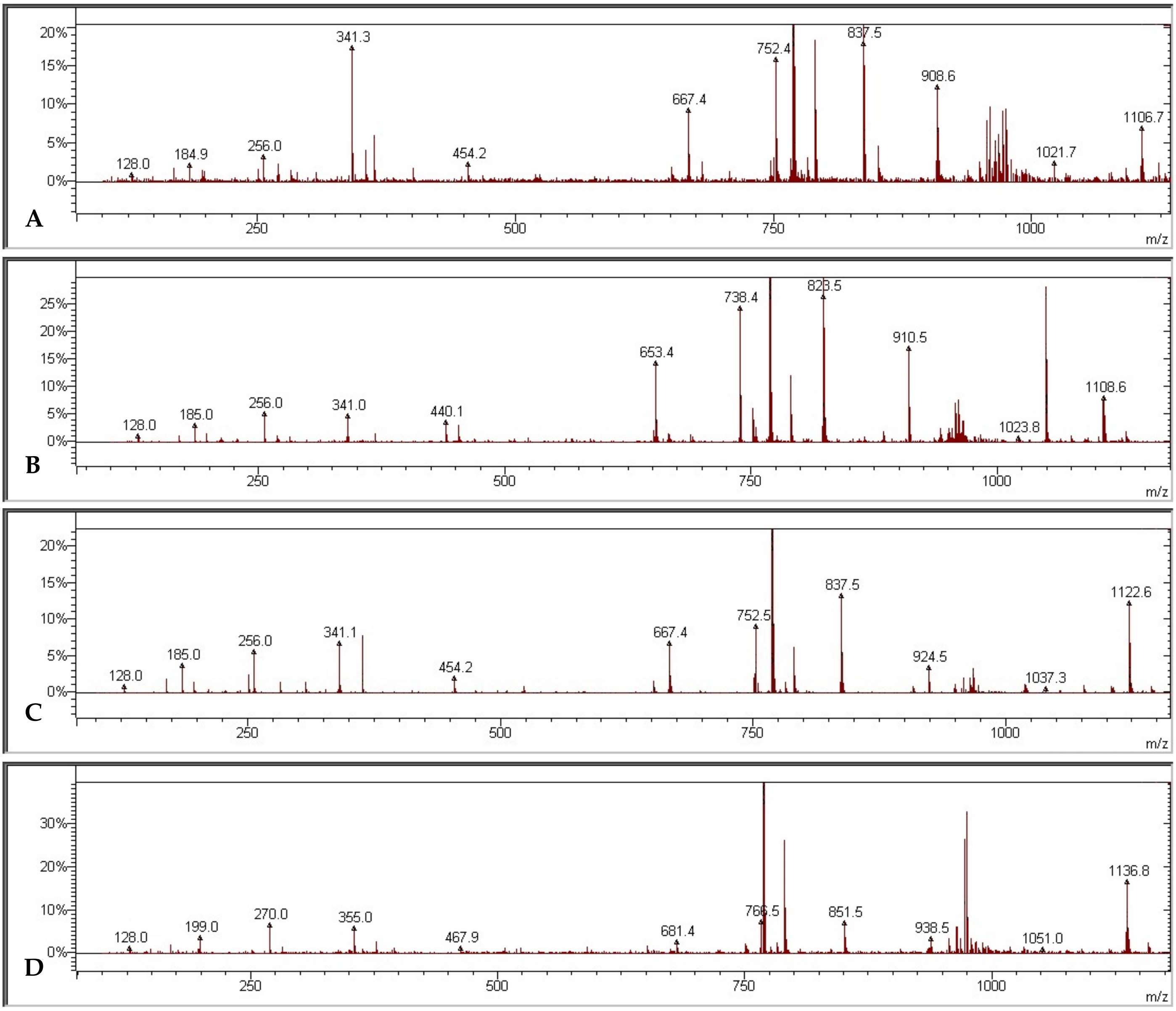

Supplementary Figure 3: Typical b ion series of described peptaibols ranged from b12 at *m/z* 1106 (A), *m/z* 1108 (B), *m/z* 1122 (C), *m/z* 1136 (D) resulting from the full scan measurements of crude peptaibol extracts prepared from *T. gamsii* SZMC 1656

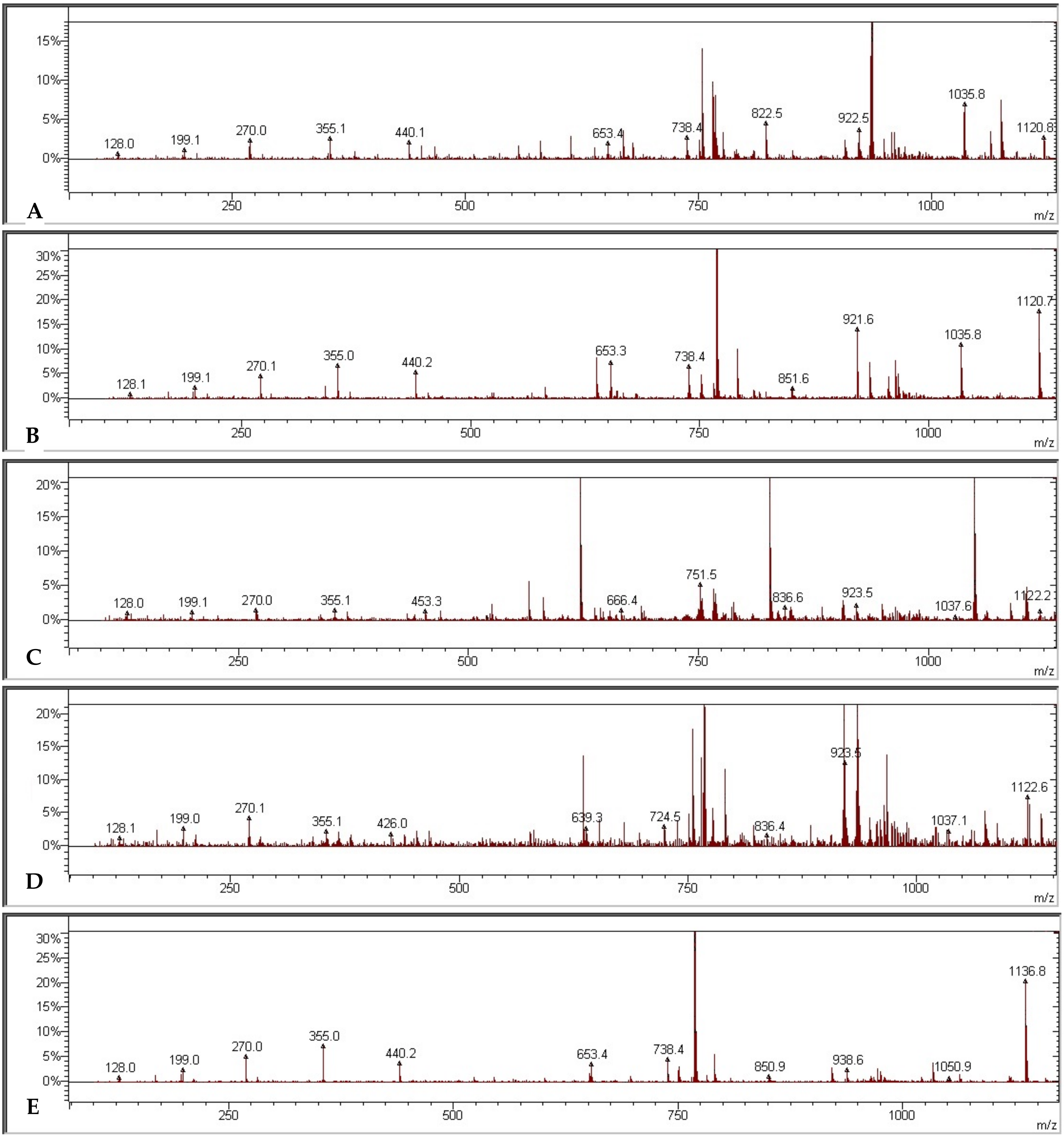

Supplementary Figure 4: Typical b ion series of described peptaibols ranging from b_{12} at *m/z* 1120 (Koningiopsin IIb, IIIb - A), *m/z* 1120 (Koningiopsins VIa, VIb - B), *m/z* 1122 (Koningiopsins Ia, Ib- C), *m/z* 1122 (Koningiopsins IIIa - D), *m/z* 1136 (Koningiopsins IIa, IV, Va, Vb - E) resulting from the full scan measurements of crude peptaibol extracts prepared from *T. koningiopsis* SZMC 12500

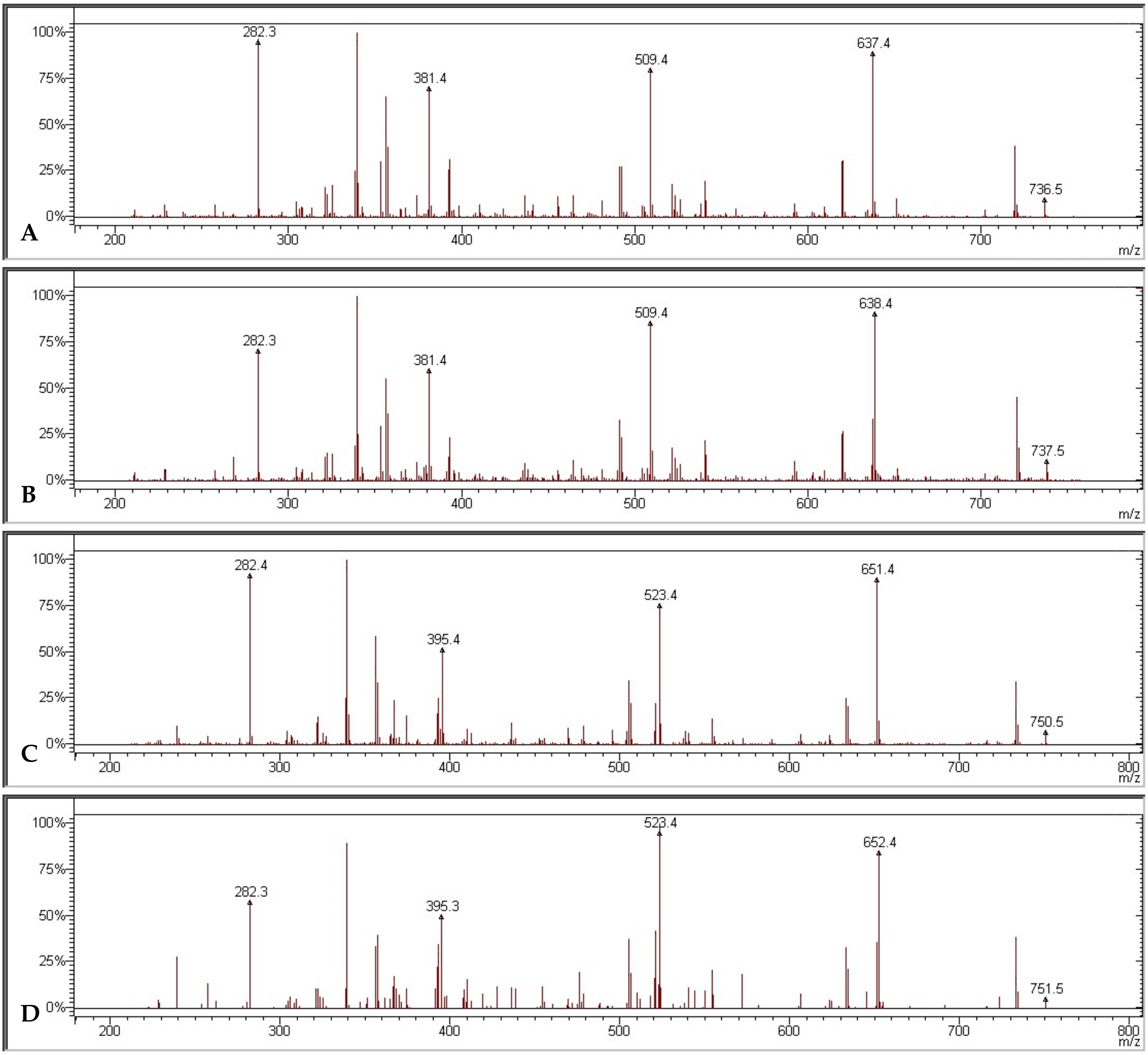

Supplementary Figure 5: Typical MS² spectra of y-ions 754.5 (A), 755.5 (B), 768.5 (C) and 769.5 (D) resulting from the full scan measurements of crude peptaibol extracts. The MS² patterns originating from the same *m*/*z* values were identical.
